# Establishing Thyroid Reference Intervals Through Hierarchical Cluster Analysis: A Comparative Evaluation of Limit Estimation and Partitioning Methods

**DOI:** 10.3390/jcm15155778

**Published:** 2026-07-23

**Authors:** Esra Yılmaz, Hülya Kılıç

**Affiliations:** 1 Clinical Biochemistry Laboratory, Karabük Training and Research Hospital, Karabuk 78200, Türkiye; trn6325@gmail.com; 2 Department of Medical Biochemistry, Faculty of Medicine, Recep Tayyip Erdogan University, Rize 53100, Türkiye

**Keywords:** artificial intelligence, data mining, hierarchical clustering, indirect method, reference values, thyroid function tests

## Abstract

**Background**: Thyroid function tests are frequently requested, but manufacturer reference intervals often lack age- or sex-based stratification. This study established indirect reference intervals for thyroid stimulating hormone (TSH), free thyroxine (fT4) and free triiodothyronine (fT3) in a large adult population. We evaluated unsupervised algorithms and conventional partitioning to identify subgroups, compared multiple limit estimation methods, and assessed the diagnostic performance of the derived intervals against an independent, clinically defined external validation cohort. **Methods**: Data from 37,255 adults (age ≥ 18) collected between 2022 and 2024 were analyzed; a reference population of 5870 individuals was established following standardized exclusion criteria. Age-based subgroups were identified through hierarchical clustering with the Elbow method, and variable importance was assessed using Random Forest analysis. Reference intervals were calculated using non-parametric, Bhattacharya, refineR, and reflimR algorithms, applied to three population frameworks: (1) the total population without stratification, (2) subgroups derived from hierarchical clustering and (3) subgroups defined by the conventional Harris–Boyd partitioning method. Diagnostic performance was subsequently evaluated in an independent external cohort (National Health and Nutrition Examination Survey [NHANES]; *N* = 2297) for all estimated reference intervals. Three classification scenarios were assessed: TSH-only, fT4-only, and combined TSH + fT4, with sensitivity, specificity, Youden index, and decision curve analysis performed for each. **Results**: Random Forest analysis identified age as the dominant variable influencing TSH, fT4 and fT3 distributions (mean decrease in accuracy: TSH 41.62, fT4 44.18, fT3 43.7), while sex showed the lowest impact. Clustering yielded six age-based subgroups for analytes. Harris–Boyd partitioning yielded six age-based subgroups for TSH, two sex-based subgroups for fT4, and six combined age-and-sex subgroups for fT3. TSH limits were broadly concordant across all three approaches (six-subgroup partitioning: 0.36–0.68 to 4.75–5.67 mIU/L). For fT4, conventional (sex-based) and clustering (age-based) partitioning produced similar ranges (11.33–20.08 pmol/L), except reflimR’s notably lower limit (10.90 pmol/L). For fT3, conventional (age + sex) and clustering (age-only) partitioning showed comparable ranges (3.36–7.03 pmol/L), with clustering revealing a clearer age-related decline in the oldest group. Diagnostic performance varied markedly by analyte. TSH-only classification achieved positive discrimination across all 13 methods (Youden index: 0.173–0.239). In contrast, fT4-only and combined TSH + fT4 classifications performed at or below chance for most methods, with 75% of the cohort falling outside fT4 reference intervals, indicating an inter-platform harmonization issue rather than a partitioning failure. Decision curve analysis confirmed TSH-only classification’s superiority, exceeding universal testing from pt ≈ 0.20 onward across all methods. **Conclusions**: Age-stratified reference intervals combined with limit estimation showed potential diagnostic advantages over manufacturer and non-stratified intervals; however, an independent external validation using a clinically defined outcome indicated that this advantage was not consistently reproduced and was dependent on the clinical decision threshold considered. These results suggest that age stratification and algorithm choice merit further clinically adjudicated validation before broad clinical adoption, and that unsupervised clustering offers a practical, objective alternative to manual subgrouping for laboratories pursuing this approach.

## 1. Introduction

Reference intervals (RIs) provide the essential statistical framework to distinguish health from disease. However, biological, environmental, and analytical variability often hinder the universal application of these intervals. For this reason, localized validation is necessary according to the Clinical and Laboratory Standards Institute (CLSI) EP28-A3c guidelines [1]. Direct approaches to establish RIs require high costs and heavy labor. To overcome these challenges, the International Federation of Clinical Chemistry and Laboratory Medicine Committee on Reference Intervals and Decision Limits (IFCC C-RIDL) advocates for indirect methods that utilize routinely collected laboratory data [2].

Thyroid disorders are prevalent endocrine conditions that disrupt physiological homeostasis. Accurate assessment of thyroid-stimulating hormone (TSH), free thyroxine (fT4), and free triiodothyronine (fT3) is indispensable for proper diagnosis and patient management [3]. Interpretive errors in these tests lead to suboptimal clinical outcomes. Therefore, the precise evaluation of thyroid function tests (TFTs) remains fundamental in clinical practice [4].

Establishing accurate RIs requires a rigorous evaluation of variance and subgroup partitioning [5]. Age and sex are standard criteria for partitioning, but other demographic and preanalytical variables may also influence laboratory results. Traditional partitioning methods often oversimplify continuous biological trends. This challenge is magnified in large-scale datasets, where population heterogeneity can easily bias calculations.

The classical approach divides data into subgroups based on traits like age, gender, and body mass index to test for significant differences. However, repeating this statistical process for every parameter is time-consuming and highly impractical. Furthermore, large data datasets can render clinically negligible differences statistically significant. Thus, objective computational approaches are essential to identify hidden patterns and capture nuanced biological variability [6,7,8].

Increased access to large-scale datasets from Laboratory Information Systems has accelerated the development of indirect RI methodologies [9]. Big data enables the application of advanced algorithms to these heterogeneous datasets [10]. When combined with clustering analysis, these datasets allow for a more effective exclusion of pathological results. Consequently, this approach helps generate laboratory-specific RIs that truly reflect real-world populations [10,11].

Advanced computational workflows and models are rapidly expanding across diverse modeling fields [12,13]. In laboratory medicine, techniques like clustering, hierarchical modeling, and ensemble methods provide robust tools. These advanced techniques identify biologically meaningful variations that are often overlooked by conventional statistics [14].

This study aims to establish reference intervals for TFTs in a large adult population using indirect methods. Specifically, it compares the subgroups identified by hierarchical clustering with those derived from conventional Harris–Boyd partitioning. Furthermore, it evaluates the performance of multiple limit estimation algorithms. Finally, it assesses the diagnostic applicability of these derived intervals through an independent, clinically defined external validation cohort.

## 2. Materials and Methods

**Figure 1:**
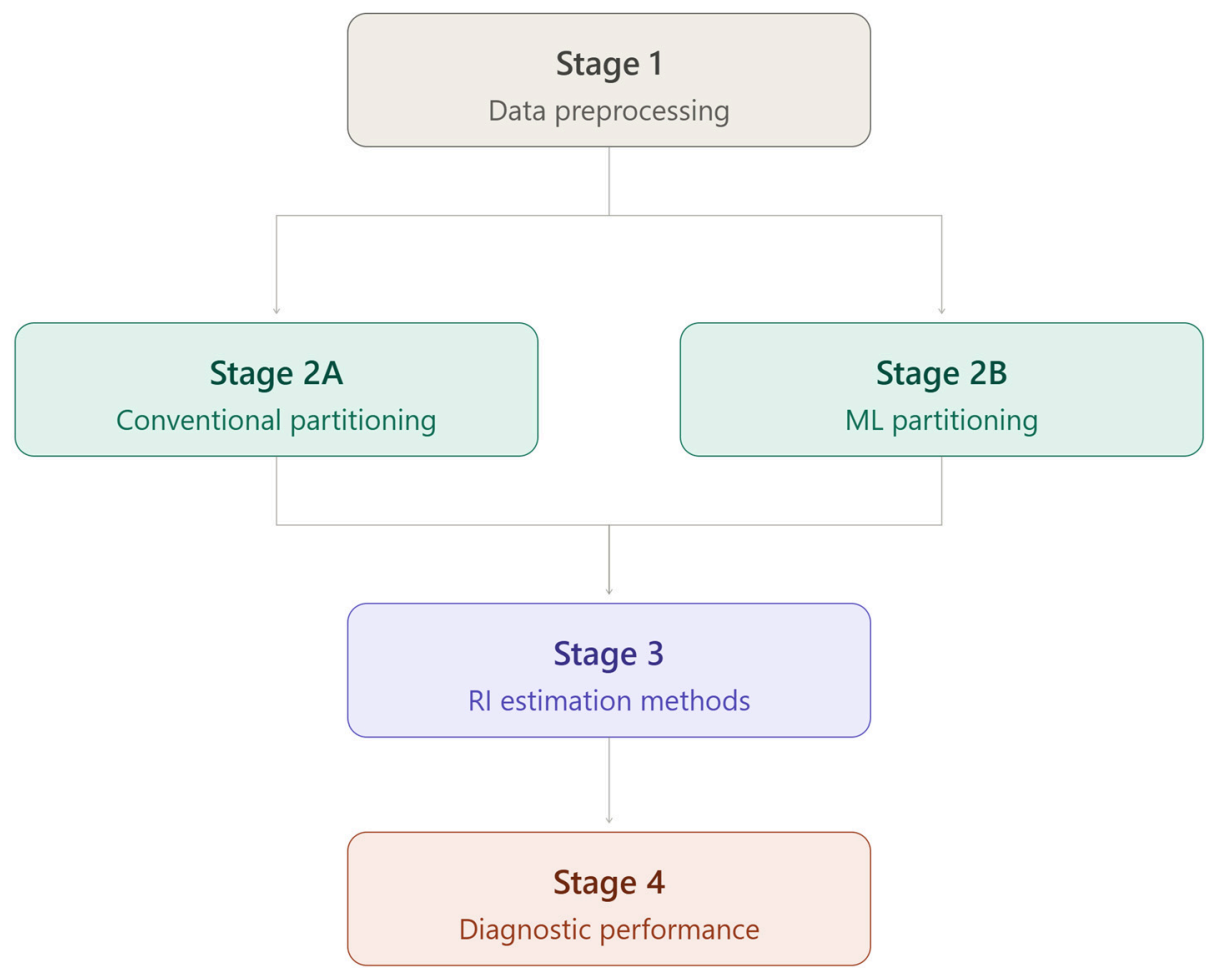
Methodological workflow for thyroid reference interval estimation.

The design and reporting of this study followed the minimum requirements for indirect reference interval determination as outlined by Jones et al. [15], including criteria for reference population selection, exclusion of pathological values, evaluation of partitioning necessity, and transparent reporting of limit estimation algorithms. The workflow chart is shown in Figure 1.

### 2.1. Sample Collection

Thyroid profiles (TSH, fT4, fT3) for outpatients aged ≥18 years were retrieved from the Laboratory Information System (LIS) (January 2022–July 2024). The samples collected only between 08:00 and 11:30 from selected non-endocrine clinics (e.g., ophthalmology, dermatology, family medicine) were included. Serum samples, collected in Vacusera (Disera, İzmir, Türkiye) gel tubes, were centrifuged at 3000× *g* for 10 min and analyzed within 3 h [16]. Measurements were performed via chemiluminescence immunoassay on the ADVIA Centaur XPT platform (Siemens, Erlangen, Germany) using manufacturer-specific reagents. Internal quality control materials were used during the study period [17,18]. Detailed analytical performance characteristics of the Siemens ADVIA Centaur XPT analyzers are provided in Supplementary Materials.

### 2.2. Data Cleaning and Selection of Reference Individuals

Reference individuals were selected from an initial pool of 97,317 results (37,255 subjects, age ≥18 years). To control for diurnal variation, only samples collected between 08:00 and 11:30 were retained [19]. Exclusion criteria included thyroid autoimmunity (anti thyroid peroxidase antibody [anti-TPO] ≥ 60 IU/mL and/or anti thyroglobulin antibody [anti-TG] ≥ 4.5 IU/mL), extreme TSH levels (≤0.1 or ≥10 mIU/L) [20,21], and comorbidities identified via 7.7 million ICD-10 records (2014–2024) [22]. Subjects with current scores ≥ 3 (A08.3-Viral enteritis, O21-Hyperemesis gravidarum) or historical scores 4–5 (E03.9-Thyrotoxicosis Hypothyroidism, C73-Thyroid malignant neoplasm) were excluded based on the 0–5 diagnostic scoring system.

**Figure 2:**
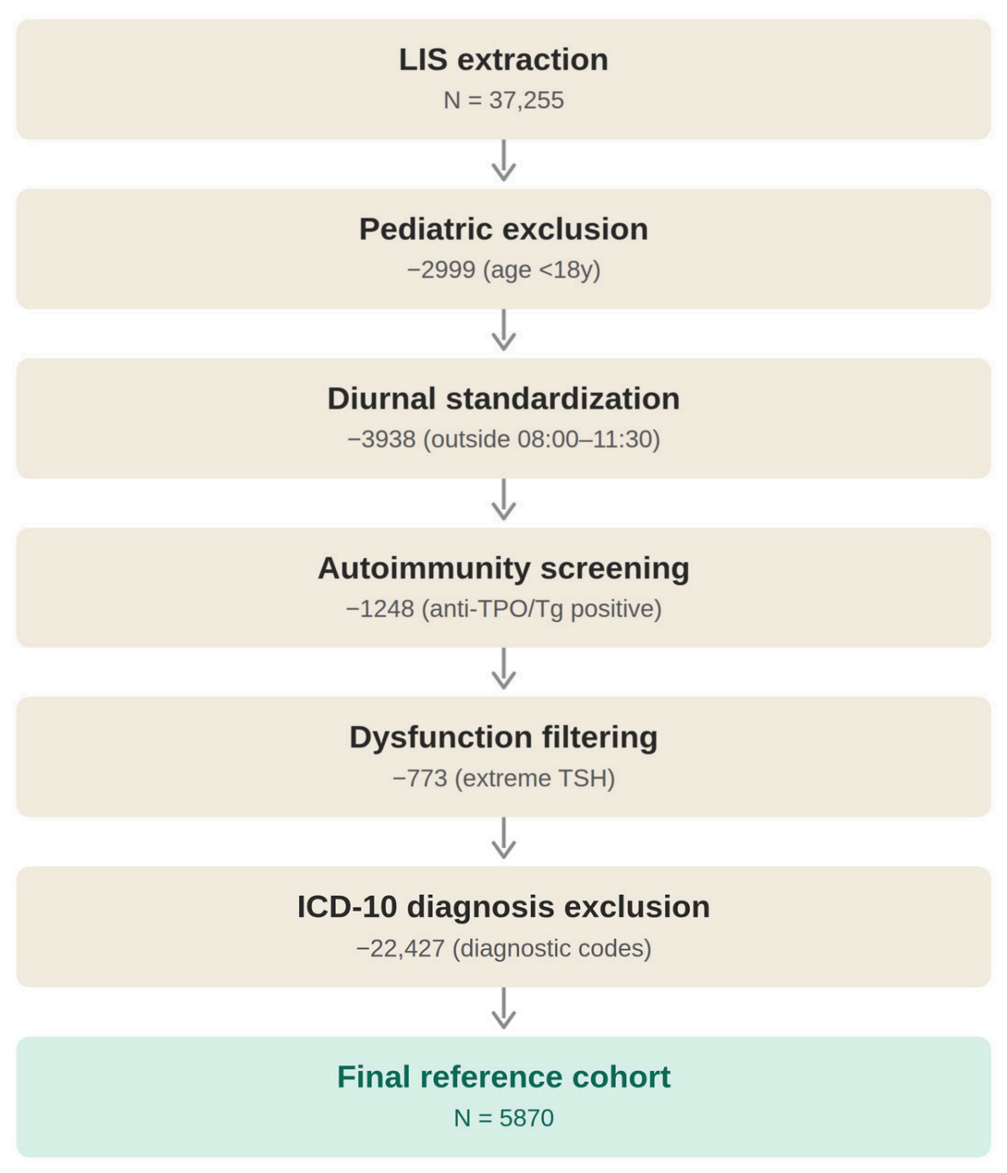
Data cleaning and selection of reference individual steps (Stage 1).

Finally, only the most recent result per individual was retained to ensure independence [15], yielding a definitive reference population of 5870 individuals. Data cleaning and selection of reference individuals are shown in Figure 2.

### 2.3. Reference Interval Estimation Workflows

#### 2.3.1. Conventional Approach

Initial assessment revealed non-normal distributions for TSH, fT4, and fT3, necessitating Box–Cox transformation. Outliers were excluded using Tukey’s method. Beyond standard age/sex categories, machine learning (ML)-derived (hierarchical clustering) age groupings were integrated into the partitioning analysis. Subgroup-specific RIs were justified via the Harris–Boyd method (Z and Z* values and standard deviations ratio) [23]. RIs were then determined using the non-parametric percentile method, with 90% confidence intervals estimated through bootstrap resampling.

#### 2.3.2. Unsupervised Machine Learning Algorithm Approach

For ML-based analyses, Box–Cox transformed data underwent Kernel Density Estimation (KDE), with a 1% density threshold applied to exclude low-density outliers and ensure clustering robustness. Subgroups were identified using agglomerative hierarchical clustering with Ward’s minimum variance method [24] and Euclidean distance. The optimal cluster count was determined via the Elbow method and visualized through dendrograms. To evaluate the relative contribution of predictors (TSH, fT4, fT3, age, and sex) to cluster formation, Random Forest models were utilized across multiple feature combinations. The importance levels of the variables were calculated in terms of Mean Decrease Accuracy (MDA) scores using the Random Forest (RF) algorithm. Fundamentally, MDA quantifies a variable’s importance by measuring how much the model’s prediction accuracy drops when that specific variable’s values are randomly permuted; a larger decrease indicates a higher reliance on that predictor for cluster identification. Each identified subgroup was analyzed independently; RIs were determined using the non-parametric percentile method, with 90% confidence intervals derived via bootstrap resampling.

Hierarchical Clustering: Used to identify natural groupings in the data by building a multilevel hierarchy of clusters [25]. Agglomerative hierarchical clustering was performed to identify natural groupings within the dataset. The dissimilarity matrix was computed using Euclidean distance, and cluster merging was executed via Ward’s minimum variance method (specifically the ‘ward.D2’ algorithm), which was selected for its capacity to minimize within-cluster variance and yield highly distinct, spherical patient subgroups. Furthermore, we specified that the Elbow method based on the Within-Cluster Sum of Squares (WSS) was used to objectively identify the optimal number of clusters for each specific analyte. To assess the reproducibility of the identified cluster structures, non-parametric bootstrap resampling (B = 1000 iterations) was performed following the framework of Hennig (2008) [26]. Cluster stability was quantified using mean Jaccard similarity indices.

Elbow Method: employed to determine the optimal number of clusters by observing the point where the rate of decrease in within-cluster variance significantly slows down [26,27].

Kernel Density Estimation: applied to visualize and estimate the continuous probability distribution of the biochemical parameters [28].

Random Forest: used to rank the clinical variables based on their contribution to the classification accuracy of the defined clusters [26,29].

#### 2.3.3. Other Indirect Approaches

Box–Cox transformation was applied prior to conventional subgrouping, clustering algorithm-assisted partitioning, Bhattacharya analysis and non-parametric RI estimation to ensure distributional normality. RefineR and reflimR algorithms were executed on filtered raw datasets.

RefineR: an advanced algorithm that estimates RIs with high accuracy by using statistical modeling to filter out pathological values from real-world data [30].

ReflimR: incorporates a three-stage algorithm based on the Hoffmann approach, providing fast and easily interpretable RIs, particularly for large datasets [31].

Bhattacharya method: A classical approach that identifies the Gaussian distribution of the healthy population by resolving the mixture distribution through graphical or mathematical decomposition [32]. RIs were additionally estimated using the Bhattacharya method (Bellview software, v2.0.1, The Netherlands) on filtered, Box–Cox-transformed datasets.

Non-parametric method: a traditional technique that determines RIs by calculating the 2.5th and 97.5th percentiles of ordered data, without assuming a normal distribution [1].

#### 2.3.4. Application of All Reference Interval Estimation Methods to All Subgroups

**Figure 3:**
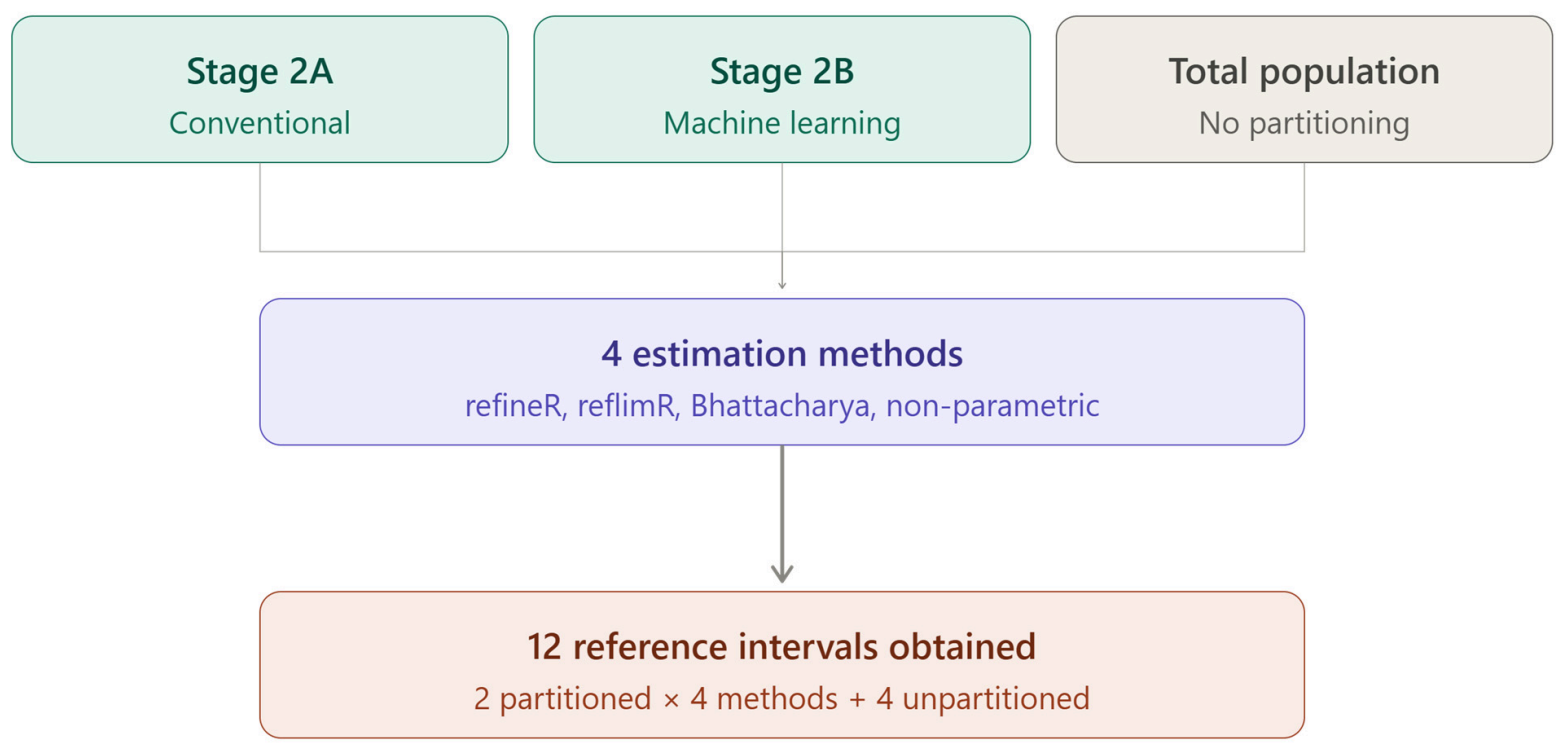
Workflow of conventional partitioning, unsupervised machine learning partitioning, and unpartitioned indirect limit estimation approaches (Stage 3).

To determine laboratory reference intervals, four limit estimation methods (refineR, non-parametric, reflimR, and Bhattacharya) were applied to all subgroups derived from unsupervised algorithms and conventional approaches. Reference interval estimations workflow (Stage 3) is shown in Figure 3.

### 2.4. Independent External Validation Cohort

To evaluate the diagnostic performance of the derived reference intervals against a clinically defined cohort, an independent external validation was performed using publicly available data from the National Health and Nutrition Examination Survey (NHANES; 2007–2008, 2009–2010, and 2011–2012 cycles). In this cohort, the patient group comprised adults reporting a current, active thyroid condition (self-reported diagnosis confirmed as still present at interview) who were not receiving thyroid-directed medication, and the reference group comprised adults reporting no history of chronic conditions; both groups excluded individuals using medications known to independently affect thyroid function tests (e.g., biotin, amiodarone, lithium, corticosteroids). The same factorial framework (machine learning versus conventional age/sex partitioning, crossed with refineR, reflimR, non-parametric, and Bhattacharya limit estimation, plus unpartitioned variants and a representative manufacturer kit-insert interval) was applied to healthy and patient cohort. Disease (non-euthyroid) status was defined as TSH alone, fT4 alone, or TSH and fT4 combined falling outside the method-specific reference interval. Diagnostic accuracy (sensitivity, specificity, Youden index) was calculated with exact 95% confidence intervals and decision curve analysis (net benefit across threshold probabilities pt = 0.01–0.50, using the binary-test extension of the standard net benefit formula, with 1000-resample bootstrap 95% confidence bands) was performed to assess clinical utility.

### 2.5. Statistical Analysis

Data extraction, organization, and partial filtering were performed using Microsoft SQL (Microsoft Corp., Redmond, WA, USA). The normality of data distribution of TSH, fT4 and fT3 was evaluated with the Kolmogorov–Smirnov test (*p* <0.001 was considered statistically significant) and outliers were excluded using the Tukey method in IBM SPSS Statistics (Version 25.0, IBM Corp., Armonk, NY, USA). The processed data were stored in Microsoft Excel 365 (Microsoft Corp., Redmond, WA, USA). After merging the Excel datasets, exploratory data analysis, data cleaning, ML algorithms, and non-parametric percentile-based reference limit calculations were all conducted using R (Version 4.4.1, R Foundation for Statistical Computing, Vienna, Austria). Analyses were performed in the RStudio environment (Version 2024.09.0 “Cranberry Hibiscus”). The diagnostic performance of the reference limits calculated by all methods, both partitioned and non-partitioned, was evaluated in the independent external validation cohort using sensitivity, specificity, and the Youden index with exact 95% confidence intervals. Clinical utility was evaluated using decision curve analysis with bootstrap-derived 95% confidence bands, performed in R (version 4.4.2).

During the preparation of this manuscript, the authors used (ChatGPT, version GPT-4/GPT-4o) for the purposes of assisting in the development of R codes for data analysis and cluster analysis modeling. The authors have reviewed and edited the output and take full responsibility for the content of this publication.

## 3. Results

### 3.1. Study Dataset and Selection of the Reference Sample

A total of 5870 individuals constituted the final reference population, comprising 3454 females (58.8%) and 2416 males (41.2%). The age range was 18–104 years in females and 18–100 years in males with an overall median age of 33 years (female: 31 years; male: 35 years). Descriptive characteristics of the reference population and analyte distributions stratified by sex are summarized in Table 1. Distributional changes before and after Box–Cox transformation are shown in Supplementary Materials Figure S1.

### 3.2. Analytical Performance

Laboratory total error (TE) rates for TSH (16.5%), fT4 (13.8%), and fT3 (12.3%) calculated using their respective CV [TSH (6.39%), fT4 (5.26%), and fT3 (4.15%)] and bias values [TSH (6.0%), fT4 (5.19%), and fT3 (5.48%)] were found to be within the CLIA-defined total allowable error (TEa) limits.

### 3.3. Reference Intervals Derived Using the Conventional Approach

Following Box–Cox transformation (sample size N for TSH, fT4 and fT3: 5829, 4456, 2033 ) and after Tukey-based outlier exclusion (sample size N for TSH, fT4 and fT3: 5759, 4414, 1995) the necessity for partitioning of subgroups derived by unsupervised algorithms was evaluated via Harris–Boyd criteria (Supplementary Materials Tables S1–S9). Comprehensive subgroup characteristics and derived limits are summarized in Table 2 (Supplementary Materials Tables S10–S12).

### 3.4. Unsupervised Machine Learning Algorithm-Derived Reference Intervals

The initial sample sizes after applying exclusion and inclusion criteria were as follows: TSH (*N =* 5829), fT4 (*N* = 4456), and fT3 (*N* = 2033). Outliers were excluded using Kernel Density Estimation (K-density). The final sample sizes were as follows: TSH (*N* = 5769), fT4 (*N* = 4411), and fT3 (*N* = 2012). Hierarchical clustering-derived reference limits for all analytes are summarized in Table 3.

Bootstrap resampling confirmed acceptable to high cluster stability across all three hormone frameworks. The fT4 model (k = 6; Table 3) demonstrated the highest reproducibility, with five of six clusters yielding mean Jaccard indices ≥0.85 (range: 0.618–0.959) and boundary uncertainty remaining low at 4.1–14.2%. The fT3 model (k = 4; Table 3) showed acceptable stability across all clusters (range: 0.663–0.832). The TSH model (k = 6; Table 3) exhibited moderate stability indices (range: 0.504–0.713), consistent with the continuous and gradual nature of age-related TSH variation. External corroboration was provided by Kayahan (2025) [14], in which K-means clustering applied to a comparable cohort independently identified an identical six-cluster age partition structure.

Subgroup characteristics are presented in Supplementary Materials Tables S13–S15. Agglomerative hierarchical clustering, optimized by the Elbow method and dendrogram inspection, was used to define objective subgroups (Supplementary Materials Figures S2 and S3). The analysis yielded six clusters for the TSH and fT4 models, and four clusters for the fT3 model. Subsequent Random Forest importance analysis was performed to identify the primary drivers for these specific clusters. The MDA scores identified age as the most influential variable across all three models, with scores of 44.2 for the fT4 model, 43.7 for the fT3 model, and 41.6 for the TSH model. In the fT3 and TSH models, the respective hormone levels followed age in importance (MDA: 16.9 and 19.7, respectively), while gender consistently showed the lowest impact on model performance across all evaluated groups. The illustration of feature importance scores for each cluster is reported in Supplementary Materials Figure S4.

### 3.5. Other Indirect Approaches

Advanced algorithms like refineR and reflimR yielded higher upper limits for TSH, while traditional methods produced narrower intervals. Although all methods showed consistency for fT4 and fT3, reflimR identified the lowest lower limit for fT4. RIs for TSH, fT4, and fT3 derived by refineR, reflimR, Bhattacharya and the non-parametric percentile method are presented in Table 4.

The details and graphical outputs of reference intervals generated by the refineR algorithm are provided in Supplementary Materials Table S16 and Supplementary Materials Figure S5. Detailed parameter estimates of the reflimR modeling process, including truncation steps and regression fits, are shown in Supplementary Materials Table S17 and Figures S6–S8. Population characteristics and detailed parameters of the non-parametric and Bhattcharya methods are presented in Supplementary Materials Tables S18 and S19 and Figure S9.

### 3.6. Application of All Reference Interval Estimation Methods to All Subgroups

Reference limits obtained from all estimation methods and subgroups are presented in Supplementary Materials Table S22. TSH upper limits demonstrated the highest variability; the *refineR* method consistently produced the narrowest upper bounds across all age groups (3.06–4.79 mIU/L), while the reflimR method yielded the widest values, exceeding the non-partitioned estimates in the 37–45 and >65 age groups. The non-parametric and Bhattacharya methods produced intermediate TSH upper limits. In contrast, TSH lower limits remained stable across methods, decreasing from younger to middle-aged groups before a partial recovery in the >65 group. Lastly, fT4 intervals were consistent across age groups and methods, except for the Bhattacharya method in the >65 group, which produced a widened interval that deviated from all other estimates.

### 3.7. Independent External Validation Cohort

In the independent Continuous NHANES (2007–2012)-derived validation cohort (*N* = 2297; 561 patients with active, medication-naïve thyroid disease and 1736 disease-free reference individuals; disease prevalence 24.4%), diagnostic performance was assessed. Baseline characteristics of the patient and reference (healthy) groups in the independent external validation cohort (NHANES, *N* = 2297) are shown in Table 5.

The retrospective evaluation of the three diagnostic scenarios yielded three critical findings that clarify the operational behavior of the reference intervals. First, under the combined TSH + fT4 criterion, diagnostic performance was remarkably modest, with all 13 methods exhibiting a negative Youden index (Supplementary Materials Table S23). Our sub-analyses revealed that this distortion was heavily driven by the fT4 component; a striking 75% of the entire cohort (including both healthy individuals and patients) fell outside the established fT4 reference intervals, underscoring severe inter-platform harmonization discrepancies. Second, when the diagnostic classification was restricted strictly to TSH alone—the analyte with the strongest and most clinically established signal for thyroid dysfunction—all 13 methods achieved positive discrimination, with Youden indices ranging from 0.173 to 0.239. Third, the fT4-only classification performed at or below chance across all frameworks, yielding negative Youden indices ranging from −0.375 to −0.266 (Supplementary Materials Table S24). This explicitly demonstrates that the overall diagnostic limitation observed in the combined panel was an artifact of fT4 performance rather than a failure of the indirect partitioning methodologies. Consequently, isolated TSH-based classification emerged as the only scientifically robust and clinically appropriate model for this cohort. Diagnostic performance of the reference interval estimation methods for TSH is shown in Table 6. Diagnostic performance of the reference interval estimation methods for TSH-fT4 combined and fT4-only is shown in Supplementary Materials Tables S23 and S24.

**Figure 4:**
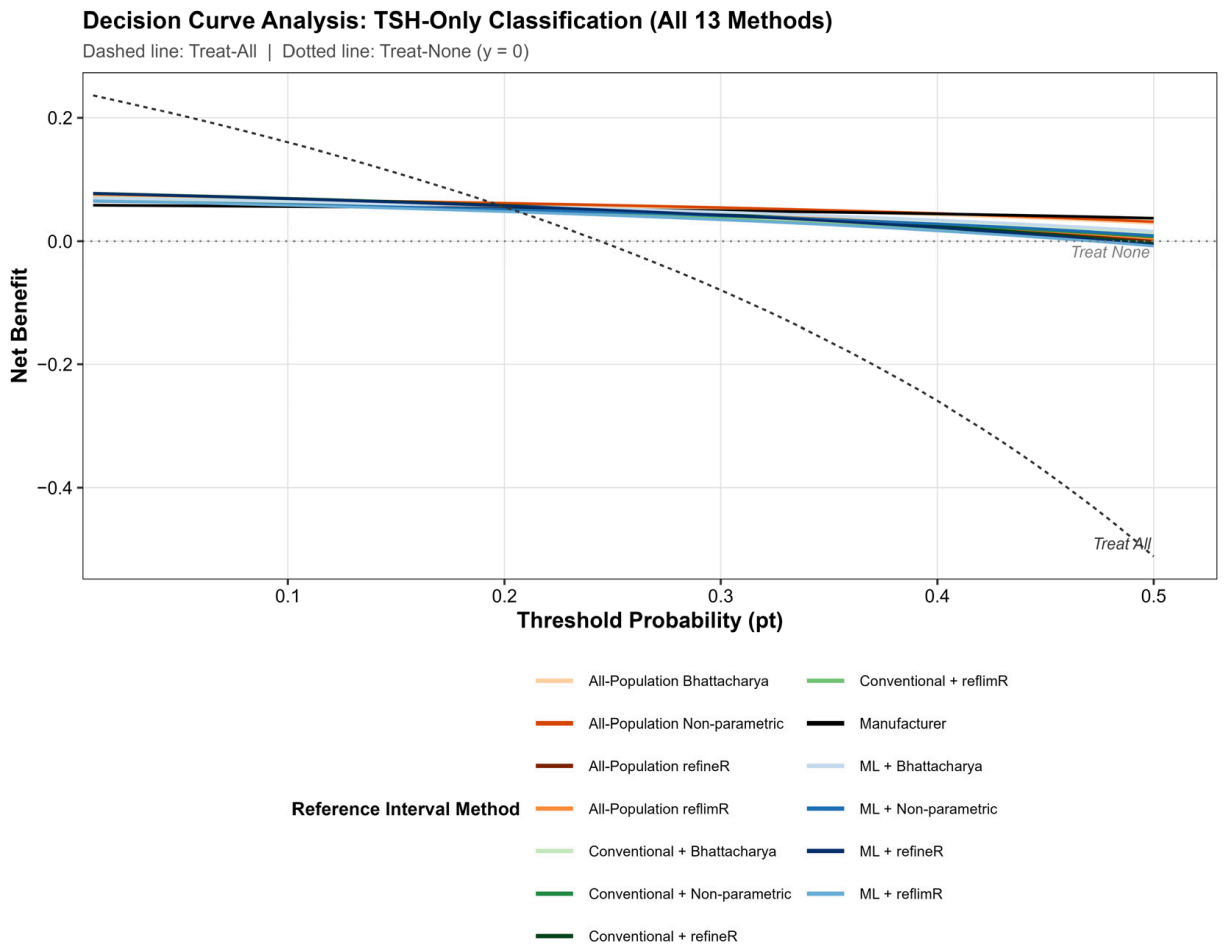
Decision curve analysis of the 13 reference interval methods for TSH.

**Figure 5:**
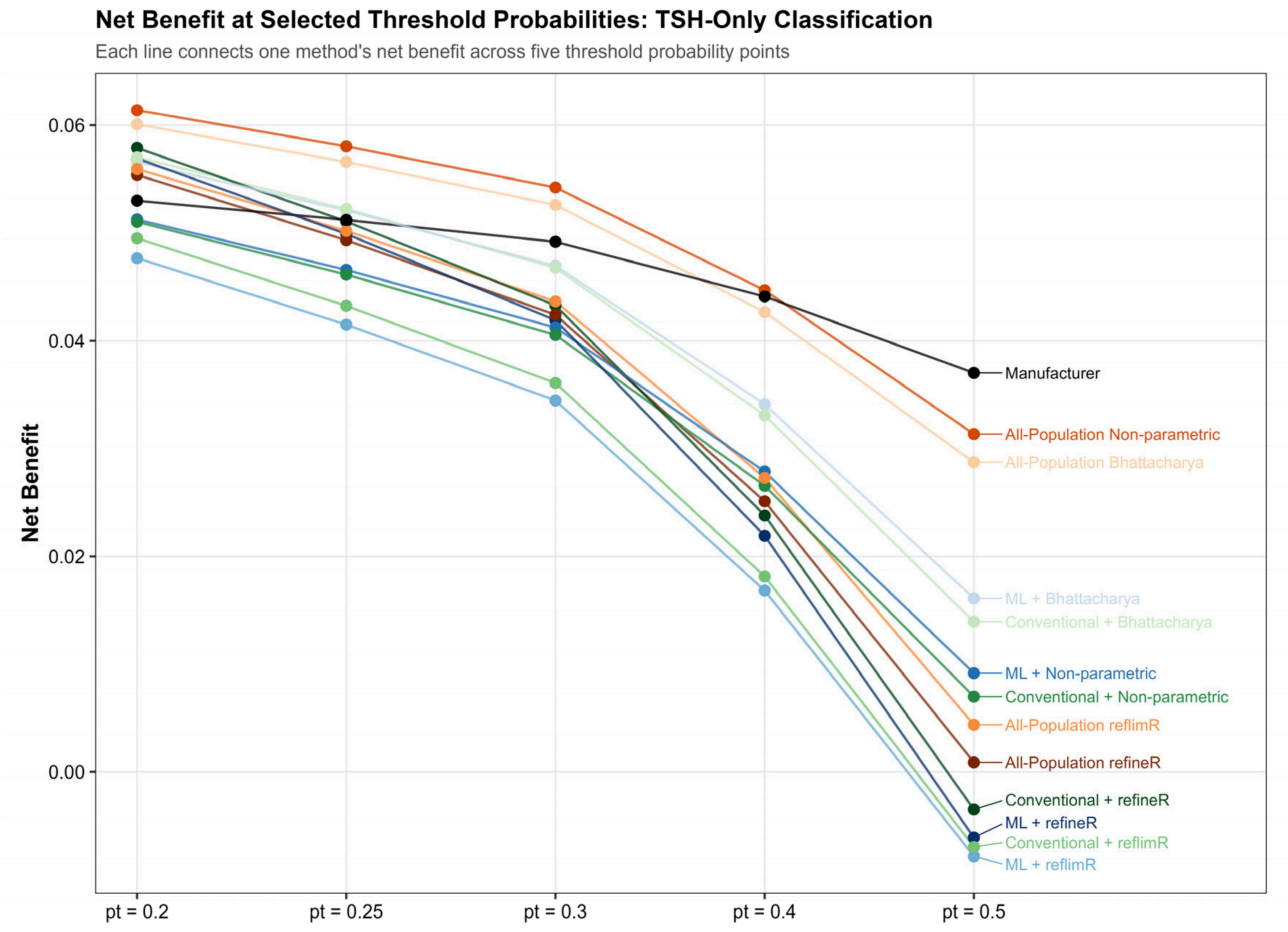
Net benefit of the 13 reference interval methods under TSH-only classification at five selected threshold probabilities (pt = 0.20, 0.25, 0.30, 0.40, 0.50).

Decision curve analysis for TSH-only classification is presented across the full threshold probability range in Figure 4, and at selected threshold probabilities for direct between-method comparison in Figure 5.

Decision curve analysis confirmed this pattern: TSH-only classification exceeded the net benefit of universal testing from pt ≈ 0.20 onward across all 13 methods, whereas the combined TSH + fT4 and fT4-only criteria required substantially higher thresholds (pt ≈ 0.25–0.40) or failed to consistently exceed treat-all, respectively (Supplementary Materials Figures S10 and S11).

### 3.8. Comparison of Derived Reference Intervals with Active and Published Reference Intervals

**Figure 6:**
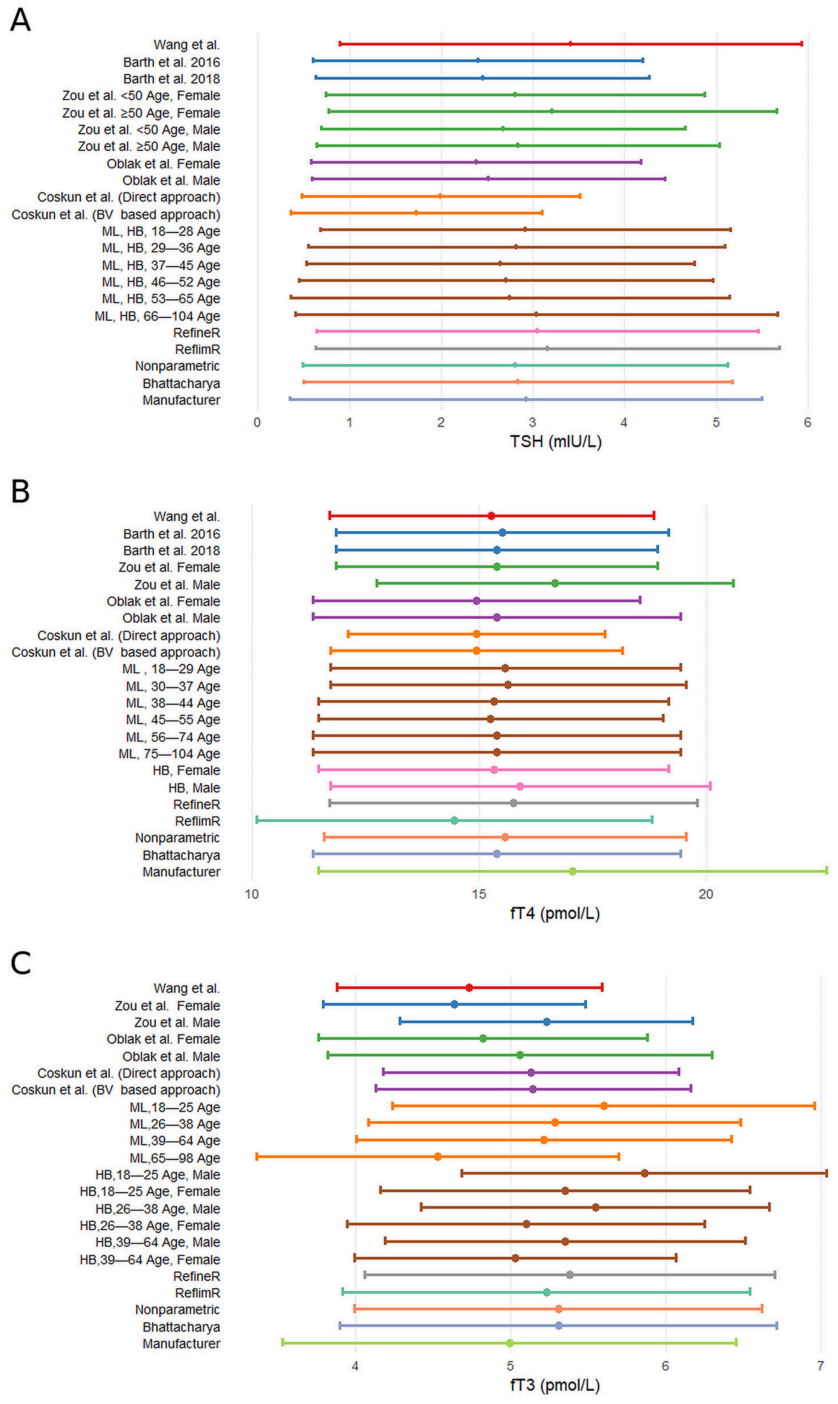
Comparison of calculated reference intervals for TSH (**A**), fT4 (**B**), and fT3 (**C**) with manufacturer limits and previous literature [33,34,35,36,37,38,39]. The figures illustrate results obtained from Bhattacharya, refineR, reflimR, non-parametric percentile method and partitioning strategies including Harris–Boyd and based hierarchical clustering. Calculated intervals are compared against the manufacturer-provided ranges and previous studies. BV: biological variation, HB: Harris–Boyd, ML: machine learning.

RIs established in this study were compared with manufacturer-provided limits and the existing literature. For structured comparison, published studies with similar methodological frameworks (indirect approach, adult populations, and comparable analytical platforms) were grouped accordingly [33,34,35,36,37,38,39]. The distribution of lower and upper reference limits for TSH, fT4 and fT3 respectively is illustrated in Figure 6.

## 4. Discussion

Our study presents a comprehensive approach that simultaneously evaluates partitioning criteria as a whole, objectively determines the hierarchical importance of these criteria, utilizes data-driven grouping methodologies, and compares the impacts of both partitioning and limit calculation methods. In addition to age and sex, which are the most common partitioning criteria, we performed clustering analysis using combinations of TSH, fT4, and fT3 to evaluate the interactions among these analytes.

The RF algorithm identified age as the most decisive variable across all clustering studies. The use of the RF algorithm in this study allowed for the objective determination of feature importance rankings [29]. When the TFT subgroups determined by agglomerative hierarchical clustering were evaluated using the conventional Harris–Boyd method, the results for fT4 were found to be inconsistent. While the same subgroups maintained their significance for TSH, an additional requirement for sex-based partitioning was identified for fT3.

In this study, the Harris–Boyd method was selected to represent the conventional approach. Since this method is optimized for normally distributed data, a Box–Cox transformation was applied to the dataset to satisfy this requirement (See Supplementary Materials Figure S1). However, the reliance of the Harris–Boyd method on Gaussian assumptions and its inherent limitations when handling more than two subgroups complicate its application in complex datasets [5]. In large-scale, real-world datasets, statistically significant differences may be observed even in the absence of a clear clinical context. Conversely, exploratory machine learning algorithms can interpret inter-individual variations by integrating multidimensional demographic data, such as sex, blood group, ethnicity, and lifestyle factors (e.g., smoking and alcohol consumption).

In our study, all analytes, along with age and sex information, were evaluated within an objective and holistic analytical framework. It must be acknowledged that the nature of statistical clustering models inherently produces precise mathematical cut-offs that do not perfectly map onto continuous biological transitions. As demonstrated in the literature by Kayahan et al. [14] using fuzzy logic modeling for thyroid reference interval estimation, biological boundaries involve dynamic and fluid transition zones rather than rigid, sharp thresholds across a population. Furthermore, the necessity of individualized reference intervals, recently emphasized by Coşkun et al. [38], underscores this inherent biological variation. The moderate boundary fluidity observed in our TSH clustering model, along with slight variations in subgroup limits across different algorithms (e.g., ages 28–29 or 45–46), may be associated with this biological reality. Therefore, these defined age partitions should not be interpreted as absolute physiological breaking points, but rather as the statistical centroids of broader endocrinological transition windows, such as early adulthood stabilization or perimenopausal and aging transitions. The integration of these data-driven subgroups into routine clinical practice and their biological validation require prospective validation studies conducted through multicenter laboratory–clinical collaborations.

It is a widely accepted clinical consensus that TSH levels physiologically increase with age; therefore, age-specific reference intervals (RIs) are a fundamental requirement for accurate diagnosis. NHANES-III data exemplified this trend, reporting TSH upper limits (97.5th percentile) of 3.5 mIU/L for ages 20–29, 4.5 mIU/L for ages 50–70, and 7.5 mIU/L for individuals over 80 years [40]. While Ehrenkranz et al. noted that seasonal variations do not significantly impact TSH rhythms, they emphasized that age, sex, time of day, and ethnicity exert substantial influences [19]. Interestingly, the same study reported that these factors did not significantly affect fT4 reference limits.

The necessity of updating RIs for older populations is further highlighted by the higher TSH upper limits observed in the elderly compared to younger adults [41]. Furthermore, sex-specific differences have been reported, with some studies finding slightly lower TSH levels in healthy men compared to women [42]. Conversely, Oblak et al. observed that while TSH increases with age in both sexes, women exhibited lower upper reference limits than men [37]. Collectively, these findings underscore the high inter-individual variation in TSH and suggest that reference limits are population-dependent, even when identical analytical methods are employed.

Regarding fT3, our findings are consistent with the existing literature suggesting that both age and sex are significant factors for partitioning [35,36,41]. Similarly, the ML-based study by Kayahan et al. identified six distinct subgroups for TSH and fT4, identifying age as the most influential factor in partitioning. Their findings are consistent with our study, specifically regarding the age-related increase in TSH upper limits and the observation of fT4 upper limits that are lower than those provided by the manufacturer [14].

In the indirect approach, the coexistence of results from both healthy and pathological individuals within the same dataset makes rigorous filtering processes essential for their effective separation [43]. The meticulous identification of truly healthy reference individuals within the dataset is a methodological necessity to enhance both analytical accuracy and model performance.

A key strength of this study is that cleansing from dirty data was performed using a rigorous and multi-layered reference individual selection protocol. The exclusion of participants exhibiting thyroid autoimmunity markers, TSH levels outside established treatment parameters, and conditions linked to thyroid dysfunction determined by a semi-quantitative ICD-10 scoring method applied from 2014 onwards enhances the precision in defining the reference population. By integrating nearly a decade of diagnostic history, potential confounding from subclinical or previously treated thyroid conditions was substantially minimized [22,44]. The final reference population of 5870 individuals, drawn from 97,317 initial test results, underscores the substantial filtering required to ensure a high-quality indirect reference cohort. The demographic profile, predominantly younger individuals with a median age of 33 years, is consistent with the outpatient clinic sources from which samples were derived, and highlights the importance of age-based partitioning, which was a consistent finding across all analytical strategies employed in this study.

RIs vary significantly based on the outlier management and population assumptions inherent to each method. The non-parametric percentile method is ideal for homogeneous groups and typically yields the narrowest intervals; however, it remains susceptible to extreme values [45]. In contrast, the Bhattacharya method isolates the predominant healthy distribution, preventing pathological data from artificially expanding the reference limits. The refineR and reflimR algorithms reflect true biological variation by correcting for distribution skewness via Box–Cox transformation. Although these methods tend to produce slightly broader intervals than conventional approaches, they exhibit high resistance to outliers [46]. Ammer et al. emphasized that refineR is highly sensitive to population characteristics; it may broaden intervals for complex parameters like TSH while maintaining limits close to conventional values for stable analytes such as fT3 and fT4 [30].

In cases of high population heterogeneity, such as wide age ranges or high disease prevalence, subgroup partitioning is critical prior to applying indirect methods; otherwise, the resulting RIs may lose clinical validity. Mirjanic-Azaric et al. showed that age-related declines in fT3 mean common reference intervals can cause false-positives in youth and false-negatives in the elderly [47].

Our evaluation of the diagnostic impact of different RI methodologies revealed that manufacturer-provided limits yielded the highest euthyroid rates. The highest hyperthyroidism rates were associated with reflimR, attributed to its lower fT4 upper limits. Conversely, the highest subclinical hyperthyroidism rates were observed with refineR and reflimR, likely due to the higher TSH lower limits generated by these approaches. These findings underscore the decisive role of method selection and population-specific (age, sex) partitioning in the clinical decision-making process. In our study, the application of various reference RI calculation methods instead of manufacturer-provided limits led to a diagnostic reclassification in 5.30 percent to 10.30 percent of the 4888 evaluated cases. This reclassification process, characterized by an upward adjustment of TSH lower limits and a downward revision of fT4 upper limits, predominantly shifted diagnoses toward the hyperthyroidism and subclinical hyperthyroidism spectrum. These findings (Supplementary Tables S20 and S21) are consistent with literature reporting a diagnostic shift of approximately 8 percent, where fewer cases were labeled as subclinical hypothyroidism and more were classified as hyperthyroidism [14,15,21].

### 4.1. Analytical Harmonization and Reference Interval Generalizability

A further explanation for the discrepant performance between TSH and fT4 relates to analytical harmonization across platforms. The IFCC Committee for Standardization of Thyroid Function Tests (C-STFT) has highlighted that, while considerable progress has been made toward harmonizing TSH measurements across commercial immunoassays, free T4 remains substantially more susceptible to inter-platform and inter-laboratory variability, owing to matrix effects and differences in antibody specificity across assay designs [48]. Consistent with this, we observed that the median fT4 value in the present validation cohort diverged considerably from the median values underlying the reference intervals evaluated—a discrepancy directly reflected in the uniformly negative Youden indices obtained when classification was restricted to fT4 alone. This pattern is consistent with a platform-level calibration mismatch rather than a limitation of the partitioning or limit estimation methods themselves. As emphasized by Ozarda et al. (2019) [49], verification of reference intervals across laboratories and analytical systems remains an unresolved challenge in laboratory medicine, and truly generalizable reference limits will likely require multicenter collaborative studies with rigorous, continuous monitoring of analytical quality indicators and harmonization against traceable reference materials [50,51]. While such harmonization efforts have been comparatively successful for TSH, free T4 standardization remains incomplete [52], and our findings derived independently from an external, population-based cohort provide empirical support for this ongoing concern.

Beyond this analyte-specific calibration issue, the effect of age/sex partitioning itself was not uniform across limit estimation algorithms. For refineR and Bhattacharya, partitioned (age/sex-stratified) methods showed higher sensitivity but lower specificity than their unpartitioned counterparts, consistent with narrower, subgroup-specific limits capturing more true cases at the cost of more false-positives. In contrast, for the non-parametric and reflimR methods, partitioned versions showed lower sensitivity together with lower specificity than the unpartitioned estimate, a pattern more consistent with reduced generalizability of subgroup-specific limits derived from smaller, stratum-level subsamples of the original derivation cohort when applied to an independent, nationally representative population, rather than a straightforward width-driven trade-off. These algorithm-specific patterns suggest that the impact of partitioning on diagnostic performance depends on the underlying estimation method and should not be generalized as a uniform effect.

### 4.2. Limitations of the Study

Several limitations should be noted. First, due to the retrospective nature of data mining, acute clinical conditions or the use of hidden medications affecting thyroid function (e.g., biotin or amiodarone) could not be identified. This retrospective approach also restricted access to clinical symptoms and definitive diagnoses. Similarly, clinicians may initiate therapy for severe TSH abnormalities even with negative autoantibodies; our inability to track these clinical decisions retrospectively limits the diagnostic performance evaluation. Second, a major limitation of the indirect method is the potential infiltration of subclinical patients into the “healthy” distribution, which can widen reference intervals, particularly the upper limits. Furthermore, age and sex stratification can reduce sample sizes in extreme populations, such as advanced age or pregnancy.

Beyond analytical and methodological considerations, several limitations specific to the design of the external validation cohort should also be acknowledged. Disease status in this cohort was ascertained through self-reported questionnaire responses rather than expert clinical adjudication, and the resulting classification was binary (presence or absence of a reported thyroid condition), without information on underlying disease subtype (e.g., autoimmune thyroiditis, nodular disease, or iatrogenic causes). Nonetheless, the detailed ascertainment of medication use—and the explicit exclusion of individuals receiving thyroid-directed therapy or medications known to interfere with thyroid function testing—strengthens the distinction between the patient and reference groups beyond what self-report alone would provide. A further limitation is that the reference (disease-free) group may have included individuals with euthyroid structural or early-stage thyroid pathology (e.g., nodules or goiter) that would not necessarily manifest as an abnormal TSH or fT4 value, as classification in this study relied exclusively on biochemical thresholds rather than clinical or imaging assessment. This may have led to an underestimation of true reference group heterogeneity and should be considered when interpreting the diagnostic performance metrics reported here.

Finally, because the analytical process strictly depends on specific instruments, assay kits, and local patient profiles, the transferability of these reference intervals to other laboratories remains limited [2,9,10,15]. Therefore, the actual utility of these new limits must be elucidated through prospective, collaborative clinical studies.

## 5. Conclusions

This study demonstrates that reference intervals for thyroid function tests can vary according to the limit estimation and partitioning strategies employed. Age-stratified reference intervals, when combined with optimized limit estimation methods, showed limited potential diagnostic advantages over manufacturer and unpartitioned intervals. However, independent external validation using a clinically defined outcome showed that this advantage was not consistently reproduced. TSH-based classification retained strong discrimination across all methods, including the manufacturer interval, whereas fT4-based classification performed poorly across all frameworks, a pattern more consistent with cross-platform harmonization differences than with a limitation of the partitioning methodology itself. Unsupervised hierarchical clustering achieved performance comparable to conventional Harris–Boyd partitioning, supporting its use as a practical and objective alternative to manual subgrouping. These findings suggest that claims of diagnostic superiority should be scoped to the specific analyte and threshold under which they were demonstrated, and that age stratification and algorithm choice require further clinically adjudicated, multicenter validation with particular attention to fT4 harmonization before broad clinical adoption.

## Figures and Tables

**Table 1:** Distribution of thyroid function test results in the reference sample population.

Analyte (Unit)	N (Female/Male)	Median (1st and 3rd Quartiles)		
		Female	Male	Total
TSH	5829	1.92	1.77	1.85
(mIU/L)	(3426/2403)	(1.30–2.83)	(1.21–2.59)	(1.26–2.73)
fT4	4456	14.54	15.44	14.93
(pmol/L)	(2551/1905)	(13.38–15.96)	(14.03–16.99)	(13.64–16.47)
fT3	2033	4.99	5.51	5.22
(pmol/L)	(1080/953)	(4.58–5.38)	(5.10–5.94)	(4.78–5.70)

Abbreviations: TSH, thyroid-stimulating hormone; fT4, free thyroxine; fT3, free triiodothyronine.

**Table 2:** Established reference intervals for thyroid parameters partitioned by the Harris–Boyd method.

	TSH			fT4			fT3		
Group	Age	Lower Limit (90% CI)	Upper Limit (90% CI)	Sex	Lower Limit (90% CI)	Upper Limit (90% CI)	Age (Sex)	Lower Limit (90% CI)	Upper Limit (90% CI)
1	18–28	0.68	5.15	Female	11.45	19.18	18–25	4.68	7.03
		(0.63–0.72)	(4.92–5.28)		(11.33–11.45)	(18.79–19.31)	(M)	(4.55–4.79)	(6.90–7.10)
2	29–36	0.59	5.06	Male	11.71	20.08	18–25	4.16	6.54
		(0.50–0.66)	(4.69–5.3)		(11.71–11.97)	(19.82–20.33)	(F)	(4.12–4.25)	(6.33–6.74)
3	37–45	0.53	4.75				26–38	4.42	6.67
		(0.46–0.59)	(4.62–5.04)				(M)	(4.21–4.53)	(6.48–6.83)
4	46–52	0.45	4.95				26–38	3.95	6.25
		(0.40–0.48)	(4.74–5.78)				(F)	(3.89–4.04)	(6.16–6.30)
5	53–65	0.38	5.14				39–64	4.19	6.51
		(0.35–0.41)	(4.82–5.65)				(M)	(4.05–4.27)	(6.42–6.62)
6	66–104	0.43	5.67				39–64	3.99	6.07
		(0.39–0.46)	(5.19–6.04)				(F)	(3.76–4.04)	(5.90–6.22)
7							65–98 *		
							(M)		
8							65–98 *		
							(F)		

* Reference limits were not calculated because the sample size was not statistically sufficient. Abbreviations: CI, confidence interval; F, female; fT4, free thyroxine; fT3, free triiodothyronine; M, male; TSH, thyroid-stimulating hormone.

**Table 3:** Unsupervised algorithm-derived age-based subgroups and reference intervals for TSH, fT4, and fT3.

	TSH			fT4			fT3		
Group	Age	Lower Limit (90% CI)	Upper Limit (90% CI)	Age	Lower Limit (90% CI)	Upper Limit (90% CI)	Age	Lower Limit (90% CI)	Upper Limit (90% CI)
1	18–28	0.68	5.15	18–29	11.71	19.43	18–25	4.24	6.96
		(0.63–0.73)	(4.94–5.3)		(11.58–11.84)	(18.79–20.21)		(4.16–4.33)	(6.88–7.05)
2	29–36	0.55	5.09	30–37	11.71	19.56	26–38	4.09	6.48
		(0.49–0.66)	(4.73–5.36)		(11.58–11.84)	(18.92–19.82)		(3.96–4.19)	(6.42–6.64)
3	37–45	0.53	4.76	38–44	11.45	19.18	39–64	4.01	6.42
		(0.46–0.60)	(4.59–5.04)		(11.33–11.71)	(18.79–19.95)		(3.86–4.11)	(6.25–6.51)
4	46–52	0.45	4.96	45–55	11.45	19.05	65–98	3.36	5.70
		(0.40–0.48)	(4.64–5.78)		(11.07–11.58)	(18.40–19.43)		(3.26–3.55)	(5.64–5.98)
5	53–65	0.36	5.14	56–74	11.33	19.43			
		(0.34–0.39)	(4.81–5.57)		(10.94–11.71)	(18.79–20.21)			
6	66–104	0.41	5.67	75–104	11.33	19.43			
		(0.32–0.44)	(5.19–6.02)		(10.94–11.71)	(18.79–19.43)			

Abbreviations: CI, confidence interval; fT4, free thyroxine; fT3, free triiodothyronine; TSH, thyroid-stimulating hormone.

**Table 4:** Reference intervals for TSH, fT4, and fT3 derived by refineR, reflimR, Bhattacharya and non-parametric percentile method.

Analyte (Unit)	Limit	RefineR (90% CI)	ReflimR (90% CI)	Non-Parametric (90% CI)	Bhattacharya (90% CI)
TSH (mIU/L)	Lower	0.64	0.63	0.49	0.50
		(0.46–0.69)	(0.61–0.65)	(0.47–0.53)	(0.48–0.51)
	Upper	5.46	5.69	5.12	5.17
		(3.39–5.75)	(5.48–5.82)	(4.99–5.22)	(5.10–5.27)
fT4 (pmol/L)	Lower	11.70	10.90	11.58	11.33
		(11.30–11.90)	(10.81–11.07)	(11.45–11.58)	(11.20–11.45)
	Upper	19.80	18.80	19.56	19.43
		(19.00–19.80)	(18.62–18.87)	(19.43–19.82)	(19.18–19.56)
fT3 (pmol/L)	Lower	4.06	3.92	3.99	3.90
		(3.94–4.23)	(3.87–3.99)	(3.92–4.09)	(3.86–3.96)
	Upper	6.70	6.54	6.62	6.71
		(6.39–6.76)	(6.46–6.59)	(6.56–6.71)	(6.65–6.77)

Abbreviations: CI, confidence interval; fT3, free triiodothyronine; fT4, free thyroxine; TSH, thyroid stimulating hormone.

**Table 5:** Baseline characteristics of the patient and reference (healthy) groups in the independent external validation cohort (NHANES, *N* = 2297).

Parameter	Patient Group (*N* = 561)	Reference Group (*N* = 1736)	*p* Value *
Age, years, mean ± SD	62.7 ± 14.6	38.5 ± 16.3	<0.001
Female sex, n (%)	443 (79.0)	672 (38.7)	<0.001
TSH, mIU/L, median (IQR)	1.78 (0.82–3.53)	1.47 (1.00–2.14)	<0.001
Free T4, pmol/L, median (IQR)	11.6 (10.1–13.4)	10.3 (9.0–11.5)	<0.001

Abbreviations: SD, standard deviation; IQR, interquartile range; TSH, thyroid-stimulating hormone. * Age was compared using Welch’s two-sample *t*-test; sex distribution was compared using Pearson’s chi-squared test with Yates’ continuity correction; TSH and fT4 were compared using the Mann–Whitney U test.

**Table 6:** Diagnostic performance of the reference interval estimation methods in the independent external TSH validation cohort.

Method	Sensitivity, % (95% CI)	Specificity, % (95% CI)	PPV, %	NPV, %	Youden Index
All-Pop. Non-parametric	29.2 (25.5–33.2)	94.7 (93.5–95.7)	64.1	80.5	+0.239
All-Pop. Bhattacharya	28.9 (25.2–32.8)	94.5 (93.3–95.5)	62.8	80.4	+0.233
Conventional + Bhattacharya	29.2 (25.5–33.2)	92.4 (91.0–93.6)	55.4	80.2	+0.216
ML + Bhattacharya	28.7 (25.0–32.6)	92.9 (91.5–94.0)	56.5	80.1	+0.216
Conventional + refineR	32.1 (28.2–36.1)	89.2 (87.6–90.6)	48.9	80.2	+0.213
Manufacturer	23.9 (20.4–27.6)	97.2 (96.3–97.9)	73.2	79.8	+0.210
All-Pop. reflimR	29.4 (26.2–33.9)	90.9 (89.4–92.2)	51.5	80.1	+0.208
ML + refineR	31.9 (28.1–35.9)	88.9 (87.3–90.3)	48.1	80.2	+0.208
All-Pop. refineR	30.1 (26.4–34.1)	90.4 (88.9–91.7)	50.3	80.0	+0.205
ML + Non-parametric	26.7 (23.1–30.6)	92.6 (91.2–93.8)	53.8	79.6	+0.193
Conventional + Non-parametric	26.9 (23.3–30.8)	92.2 (90.9–93.4)	52.8	79.6	+0.191
Conventional + reflimR	28.0 (24.3–31.9)	90.0 (88.5–91.4)	47.6	79.5	+0.180
ML + reflimR	27.1 (23.5–31.0)	90.2 (88.7–91.6)	47.2	79.3	+0.173

Abbreviations: PPV, positive predictive value; NPV, negative predictive value; CI, confidence interval; ML, machine learning (data-driven partitioning); All-Pop., All-Population (unpartitioned). Methods are ordered by descending Youden index within each table. *N =* 561 patients, *N =* 1736 reference individuals; disease prevalence = 24.4%.

## Data Availability

The codes used for data analysis and clustering analysis are archived on Zenodo. During the peer-review process, these codes are available to reviewers via a private access link (https://zenodo.org/records/19701443?token=eyJhbGciOiJIUzUxMiJ9.eyJpZCI6IjVmOTA0NzEzLTBmYTctNGEyYi1iY2QxLThmODgxZjdlMDI3NSIsImRhdGEiOnt9LCJyYW5kb20iOiI5NWVkNGYzNDdmNmY2MGE2MjhjZDgzODMxOWJhOTJhNiJ9.Y07jKQy3Etfzv2dIP65tXDKC7px0zer_EAekIFHU4we_hMiM6W83Tpgw1cb1VrLEiGBzWRHliHzLCiCh9uvUzg [accessed on 19 July 2026]) (DOI: https://doi.org/10.5281/zenodo.19701443). Due to privacy regulations, the original dataset cannot be shared. The data available on Zenodo is a synthetic version designed to mimic the statistical properties of the original. The original data presented in this study are available on request from the corresponding author.

## Supplementary Materials

The following supporting information can be downloaded at: https://www.mdpi.com/article/10.3390/jcm15155778/s1.

## Abbreviations

The following abbreviations are used in this manuscript:

RIs	reference intervals
CLSI	Clinical and Laboratory Standards Institute
IFCC	International Federation of Clinical Chemistry and Laboratory Medicine
C-RIDL	Committee on Reference Intervals and Decision Limits
TSH	thyroid-stimulating hormone
fT4	free thyroxine
fT3	free triiodothyronine
TFTs	thyroid function tests
HC	hierarchical clustering
LIS	Laboratory Information System
anti-TPO	Anti-Thyroid Peroxidase Antibodies
anti-TG	Anti-Thyroglobulin Antibodies
KDE	Kernel Density Estimation
MDA	Mean Decrease Accuracy
NHANES	National Health and Nutrition Examination Survey
TE	Total Error
TEa	Allowable Total Error
RF	Random Forest
