# Supplementary material for: Establishing Thyroid Reference Intervals Through Hierarchical Cluster Analysis: A Comparative Evaluation of Limit Estimation and Partitioning Methods
Source: J Clin Med. 2026 Jul 23;15(15):5778. doi: 10.3390/jcm15155778 (PMC13467166; doi:10.3390/jcm15155778)
Supplement: Supplementary file 1 [file jcm-15-05778-s001.zip › jcm-4354748-supplementary.pdf]

Evaluation of Analytical Performance

The analytical performance of TSH, fT4 and fT3 measurements was evaluated. All assays were performed on the Siemens ADVIA Centaur XPT analyzer (Siemens Healthineers, Erlangen, Germany) using chemiluminescent immunoassay (CLIA) technology with manufacturer-specific reagents and calibrators.

Internal quality control (was conducted using multi-level Randox materials (Randox Laboratories, UK), with TSH evaluated at three levels and fT4 and fT3 at two levels. The pooled analytical coefficient of variation (CV) was calculated from a comprehensive dataset (TSH: n=469; fT3: n=1,174; fT4: n=3,853). Systematic error (bias) was derived from 24 months of external quality assessment (EQA) data via the RIQAS Immunoassay Program and peer-group reports. Total analytical error (TAE) was determined using the formula:

$$\%\mathrm{TAE} = \%\mathrm{Bias} + 1.65 \times \%\mathrm{CV}$$

Analytical performance was benchmarked against the Clinical Laboratory Improvement Amendments (CLIA '25) guideline. For fT3, where CLIA '25 targets are undefined, the New York State Department of Health (NYS) Clinical Laboratory Evaluation Program criteria (±25%) were adopted. All parameters demonstrated high proficiency, consistently meeting the predefined Total Allowable Error (TEa) requirements.

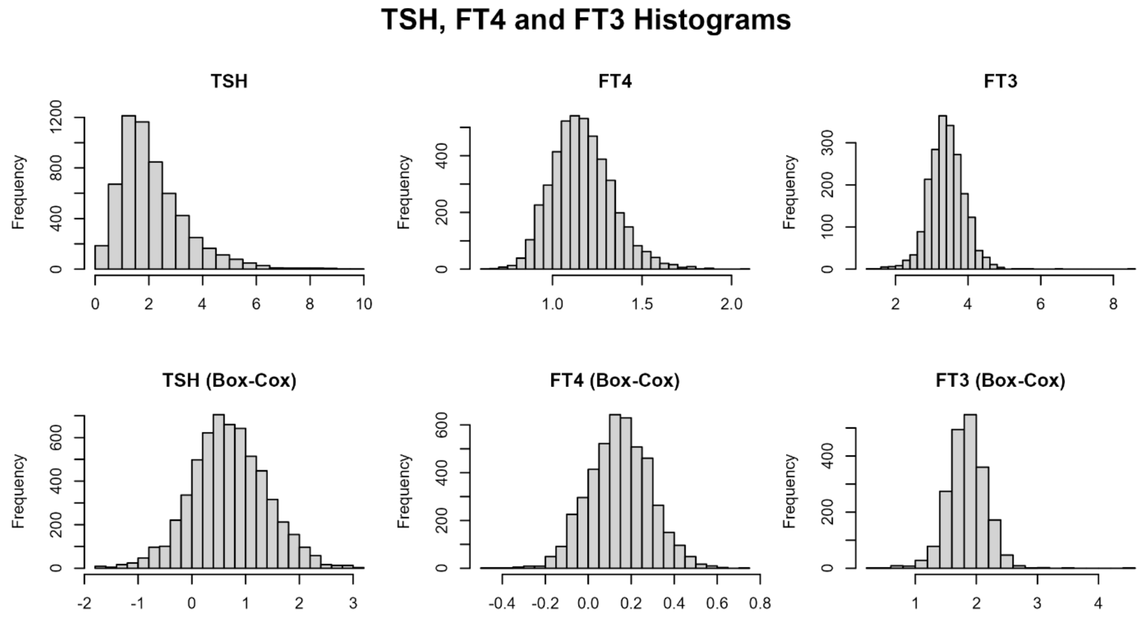

Figure S1. Histograms of original and Box–Cox–transformed TSH, fT4, and fT3 data after application of filtering procedures, without outlier exclusion. Abbreviations: FT3, free triiodothyronine; FT4, free thyroxine; TSH, thyroid stimulating hormone.

Table S1. Z and Z* values (Z/Z*) for TSH age subgroups

Age Group	18–28	29–36	37–45	46–52	53–65
29–36	1.04 / 3.18				
37–45	2.25 / 3.06	**3.52 / 3.24**			
46–52	1.59 / 2.94	0.84 / 3.12	**3.56 / 3.00**		
53–65	**3.37 / 3.12**	2.78 / 3.30	**5.43 / 3.18**	1.50 / 3.06	
66–104	1.82 / 2.87	1.15 / 3.06	**3.65 / 2.94**	0.33 / 2.81	1.05 / 3.00

Z-value: Represents the calculated statistical difference between the means of two subgroups. Z*-value: Represents the calculated threshold based on the sample sizes and standard deviations of the groups. Values in **bold** indicate pairs where Z> Z*, meeting the statistical criteria for partitioning according to the Harris-Boyd method ($p < 0.05$).
Abbreviations: TSH, thyroid stimulating hormone.

Table S2. Standard deviation ratios for TSH age subgroups

Age Group	18–28	29–36	37–45	46–52	53–65
29–36	1.18				
37–45	0.92	0.77			
46–52	0.84	0.71	0.92		
53–65	0.84	0.71	0.91	0.99	
66–104	0.78	0.66	0.85	0.93	0.93

According to Harris-Boyd criteria, a standard deviation ratio≥ 1.5 indicates significant non-homogeneity between subgroups, necessitating separate reference intervals for clinical accuracy, even if the Z-test criteria are not met.
Abbreviations: TSH, thyroid stimulating hormone.

Table S3. Z/Z* values and standard deviation ratios for TSH sex subgroups

Sex	Z / Z*	Standard Deviation Ratio
Female / Male	5.89 / 14.69	1.01

Z-value: Represents the calculated statistical difference between the means of two subgroups. Z*-value: Represents the calculated threshold based on the sample sizes and standard deviations of the groups. According to Harris-Boyd criteria, a standart deviation ratio≥ 1.5 indicates significant non-homogeneity between subgroups, necessitating separate reference intervals for clinical accuracy, even if the Z-test criteria are not met.
Abbreviations: TSH, thyroid stimulating hormone.

Table S4. Z and Z* values (Z/Z*) for fT4 age subgroups

Age Group	18–28	30–37	38–44	45–55	56–74
30–37	2.33 / 9.80				
38–44	3.00 / 9.55	0.70 / 6.41			
45–55	6.61 / 9.92	3.38 / 6.95	2.48 / 6.59		
56–74	3.95 / 9.71	1.46 / 6.66	0.71 / 6.41	1.75 / 6.83	
75–104	3.30 / 8.81	2.27 / 5.25	1.89 / 4.76	0.69 / 5.46	1.53 / 5.09

Z-value: Represents the calculated statistical difference between the means of two subgroups. Z*-value: Represents the calculated threshold based on the sample sizes and standard deviations of the groups.
Abbreviations: fT4, free thyroxine.

Table S5. Standard deviation ratios for fT4 age subgroups

Age Group	18–28	30–37	38–44	45–55	56–74
30–37	1.00				
38–44	1.01	1.01			
45–55	1.01	1.01	1.03		
56–74	1.05	1.04	1.06	1.03	
75–104	1.25	1.25	1.27	1.23	1.19

According to Harris-Boyd criteria, a standart deviation ratio≥ 1.5 indicates significant non-homogeneity between subgroups, necessitating separate reference intervals for clinical accuracy, even if the Z-test criteria are not met.
Abbreviations: fT4, free thyroxine.

Table S6. Z/Z* values and standard deviation ratios for fT4 sex subgroups

Sex	Z / Z*	Standard Deviation Ratio
Female / Male	**13.02/12.86**	1.03

Z-value: Represents the calculated statistical difference between the means of two subgroups. Z*-value: Represents the calculated threshold based on the sample sizes and standard deviations of the groups. According to Harris-Boyd criteria, a standart deviation ratio≥ 1.5 indicates significant non-homogeneity between subgroups, necessitating separate reference intervals for clinical accuracy, even if the Z-test criteria are not met. Values in **bold** indicate pairs where Z> Z*, meeting the statistical criteria for partitioning according to the Harris-Boyd method (p <0.05).
Abbreviations: fT4, free thyroxine.

Table S7. Z and Z* values (Z/Z*) for fT3 age subgroups

Age Group	18–25	26–38	39–64
26–38	4.63 / 6.54		
39–64	**9.93 / 7.01**	4.95 / 6.80	
65–98	**15.91 / 5.40**	**12.78 / 5.12**	**10.00 / 5.71**

Z-value: Represents the calculated statistical difference between the means of two subgroups. Z*-value: Represents the calculated threshold based on the sample sizes and standard deviations of the groups. Values in **bold** indicate pairs where Z> Z*, meeting the statistical criteria for partitioning according to the Harris-Boyd method ($p < 0.05$).
Abbreviations: fT3, free triiodothyronine.

Table S8. Standard deviation ratios for fT3 age subgroups

Age Group	18–25	26–38	39–64
26–38	1.08		
39–64	1.12	1.03	
65–98	1.06	1.02	1.06

According to Harris-Boyd criteria, a standart deviation ratio≥ 1.5 indicates significant non-homogeneity between subgroups, necessitating separate reference intervals for clinical accuracy, even if the Z-test criteria are not met.
Abbreviations: fT3, free triiodothyronine.

Table S9. Z/Z* values and standard deviation ratios for fT3 sex subgroups

Sex	Z / Z*	Standard Deviation Ratio
Female / Male	**16.80/8.64**	1.07

Z-value: Represents the calculated statistical difference between the means of two subgroups. Z*-value: Represents the calculated threshold based on the sample sizes and standard deviations of the groups. According to Harris-Boyd criteria, a standart deviation ratio≥ 1.5 indicates significant non-homogeneity between subgroups, necessitating separate reference intervals for clinical accuracy, even if the Z-test criteria are not met. Values in **bold** indicate pairs where Z> Z*, meeting the statistical criteria for partitioning according to the Harris-Boyd method (p <0.05).
Abbreviations: fT3, free triiodothyronine.

Table S10. Age ranges, sample sizes, median, interquartile ranges and reference limits for the six TSH groups determined by the Harris and Boyd method.

Group	Age Range	Median	1st Quartile (Q1)	3rd Quartile (Q3)	Female (N)	Male (N)	Lower Limit (90% CI)	Upper Limit (90% CI)
1	18 – 28	1.99	1.40	2.79	1503	857	0.68 (0.63-0.72)	5.15 (4.92-5.28)
2	29 – 36	1.91	1.34	2.70	471	392	0.59 (0.50-0.66)	5.06 (4.69-5.3)
3	37 – 45	1.82	1.26	2.75	317	232	0.53 (0.46-0.59)	4.75 (4.62-5.04)
4	46 – 52	1.80	1.14	2.80	398	407	0.45 (0.40-0.48)	4.95 (4.74-5.78)
5	53 – 65	1.53	1.01	2.42	480	357	0.38 (0.35-0.41)	5.14 (4.82-5.65)
6	66 – 104	1.56	1.01	2.66	223	131	0.43 (0.39–0.46)	5.67 (5.19–6.04)

TSH Unit: mIU/L
Abbreviations: CI, Confidence interval; TSH, thyroid stimulating hormone.

Table S11. Age ranges, sample sizes, median, interquartile ranges (ng/dL), and reference limits for the two fT4 groups determined by the Harris and Boyd method.

Group	Sex (N)	Median	1st Quartile (Q1)	3rd Quartile (Q3)	Age Range	Lower Limit (90% CI)	Upper Limit (90% CI)
1	Female (2528)	14.54	13.38	15.96	18 – 104	11.45 (11.33–11.45)	19.18 (18.79–19.31)
2	Male (1885)	15.44	14.03	16.99	18 – 100	11.71 (11.71–11.97)	20.08 (19.82–20.33)

TSH Unit: mIU/L,

Abbreviations: CI, Confidence interval; fT4, free thyroxine.

Table S12. Age ranges, sample sizes, median, and interquartile ranges (pg/mL) for the eight fT3 groups determined by the Harris and Boyd method, including reference limits.

Group	Age Range	Sex (N)	Median	1st Quartile (Q1)	3rd Quartile (Q3)	Lower Limit (90% CI)	Upper Limit (90% CI)
1	18 – 25	Male (229)	5.90	5.53	6.30	4.68 (4.55-4.79)	7.03 (6.90-7.10)
2	18 – 25	Female (376)	5.22	4.79	5.62	4.16 (4.12-4.25)	6.54 (6.33-6.74)
3	26 – 38	Male (291)	5.59	5.21	5.94	4.42 (4.21-4.53)	6.67 (6.48-6.83)
4	26 – 38	Female (265)	4.98	4.68	5.35	3.95 (3.89-4.04)	6.25 (6.16-6.30)
5	39 – 64	Male (363)	5.35	5.01	5.70	4.19 (4.05-4.27)	6.51 (6.42-6.62)
6	39 – 64	Female (336)	4.93	4.52	5.27	3.99 (3.76-4.04)	6.07 (5.90-6.22)
7	65 – 98	Male (71)	4.72	4.39	5.15	*	*
8	65 – 98	Female (89)	4.55	4.27	4.87	*	*

fT3 Unit: pmol/L,

Abbreviations: CI, Confidence interval; fT3, free triiodothyronine.

*Reference limits were not calculated because the sample size was not statistically sufficient.

Table S13. Age ranges, sample sizes, median, and interquartile ranges (mIU/L) for the six groups determined by hierarchical clustering using age, gender, and TSH criteria, including reference limits.

Group	Age Range	Median	1st Quartile (Q1)	3rd Quartile (Q3)	Female (N)	Male (N)	Lower Limit (90% CI)	Upper Limit (90% CI)
1	18 – 28	1.99	1.40	2.79	1503	857	0.68 (0.63-0.73)	5.15 (4.94-5.3)
2	29 – 36	1.92	1.34	2.69	470	390	0.55 (0.49-5.36)	5.09 (4.73-5.36)
3	37 – 45	1.82	1.26	2.75	398	407	0.53 (0.46-0.60)	4.76 (4.59-5.04)
4	46 – 52	1.80	1.14	2.80	317	232	0.45 (0.40-0.48)	4.96 (4.64-5.78)
5	53 – 65	1.54	1.03	2.43	478	355	0.36 (0.34-0.39)	5.14 (4.81-5.57)
6	66 – 104	1.56	1.02	2.66	220	131	0.41 (0.32-0.44)	5.67 (5.19-6.02)

Abbreviations: CI, Confidence interval; TSH, thyroid stimulating hormone.

Table S14. Age ranges, sample sizes, median, and interquartile ranges (pmol/L) for the six fT4 groups determined by hierarchical clustering, including reference limits.

Group	Age Range	Median	1st Quartile (Q1)	3rd Quartile (Q3)	Female (N)	Male (N)	Lower Limit (90% CI)	Upper Limit (90% CI)
1	18 – 29	15.32	13.77	16.99	1230	720	11.71 (11.58–11.84)	19.43 (18.79–20.21)
2	30 – 37	15.19	13.64	16.60	314	300	11.71 (11.58–11.84)	19.56 (18.92–19.82)
3	38 – 44	14.93	13.51	16.60	222	261	11.45 (11.33–11.71)	19.18 (18.79–19.95))
4	45 – 55	14.67	13.26	16.22	368	306	11.45 (11.07–11.58)	19.05 (18.40–19.43)
5	56 – 74	14.93	13.38	16.60	301	267	11.33 (10.94–11.71)	19.43 (18.79–20.21))
6	75 – 104	14.16	13.00	16.34	91	30	11.33 (10.94–11.71)	19.43 (18.79–19.43)

Abbreviations: CI, Confidence interval; fT4, free thyroxine.

Table S15. Age ranges, sample sizes, median, and interquartile ranges (pmol/L) for the four fT3 groups determined by hierarchical clustering, including reference limits.

Group	Age Range	Median	1st Quartile (Q1)	3rd Quartile (Q3)	Female (N)	Male (N)	Lower Limit (90% CI)	Upper Limit (90% CI)
1	18 – 25	5.50	4.99	5.97	377	233	4.24 (4.16–4.33)	6.96 (6.88–7.05)
2	26 – 38	5.30	4.87	5.76	252	279	4.09 (3.96–4.19)	6.48 (6.42–6.64)
3	39 – 64	5.13	4.73	5.51	338	364	4.01 (3.86–4.11)	6.42 (6.25–6.51)
4	65 – 98	4.58	4.25	5.01	97	71	3.36 (3.26–3.55)	5.70 (5.64–5.98)

Abbreviations: CI, Confidence interval; fT3, free triiodothyronine.

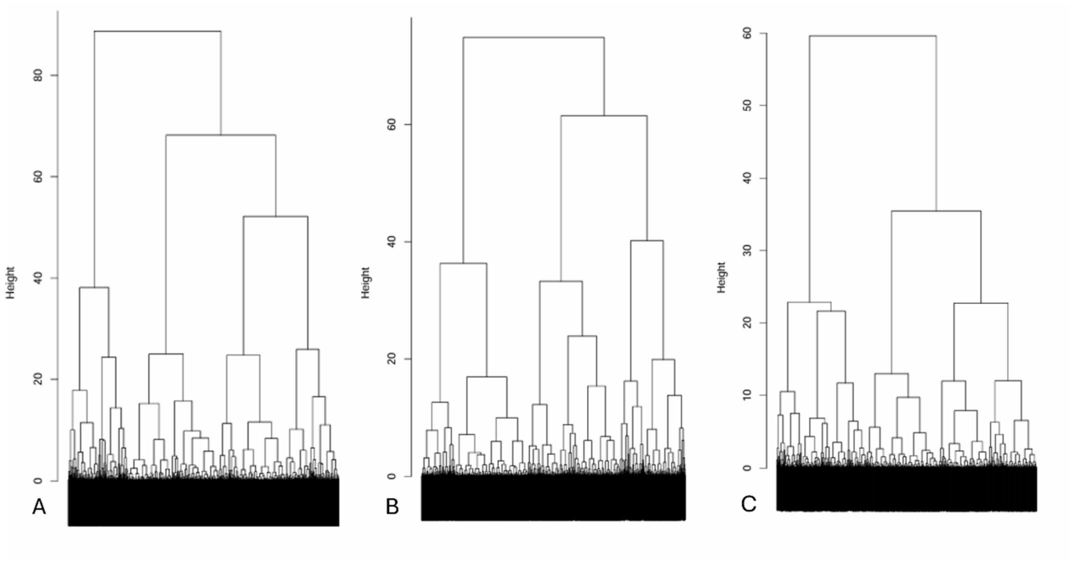

Figure S2. Dendrograms representing the results of agglomerative hierarchical clustering analysis for TSH (A), fT4 (B), and fT3 (C) parameters. The dendrogram illustrates the hierarchical clustering of data points based on their similarities; the vertical axis represents the dissimilarity level at which clusters are merged, where lower nodes indicate higher similarity between individual observations. Abbreviations: fT3, free triiodothyronine; fT4, free thyroxine; TSH, thyroid stimulating hormone.

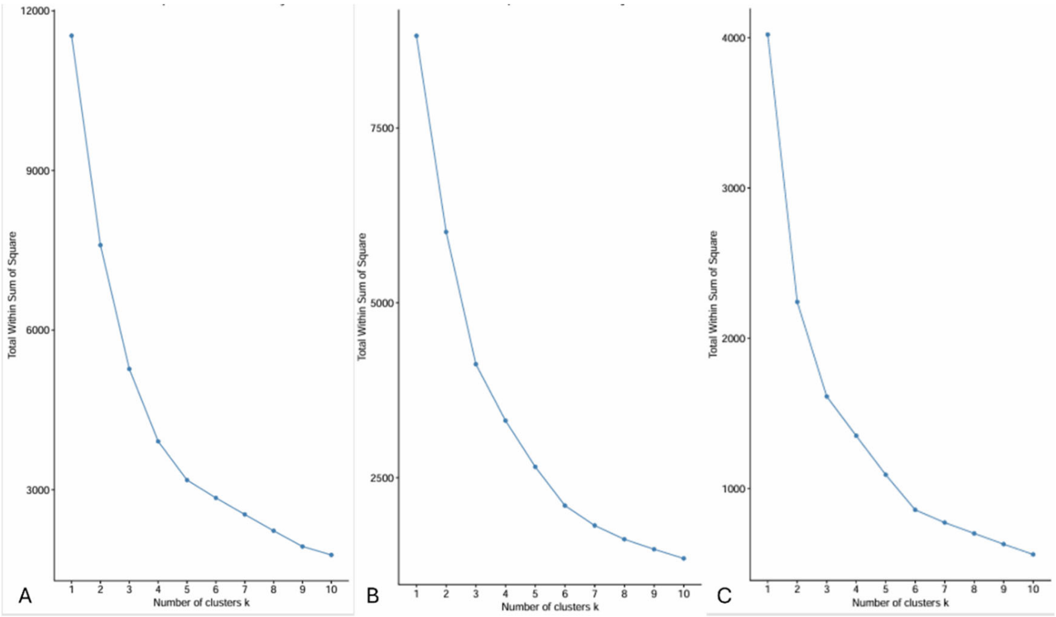

Figure S3. Selection of the optimal cluster counts (k) for TSH (A), fT4 (B), and fT3 (C) using the Elbow method. The Elbow method identifies the optimal number of clusters by locating the point where adding another cluster does not provide a significantly better fit to the data. Abbreviations: fT3, free triiodothyronine; fT4, free thyroxine; TSH, thyroid stimulating hormone.

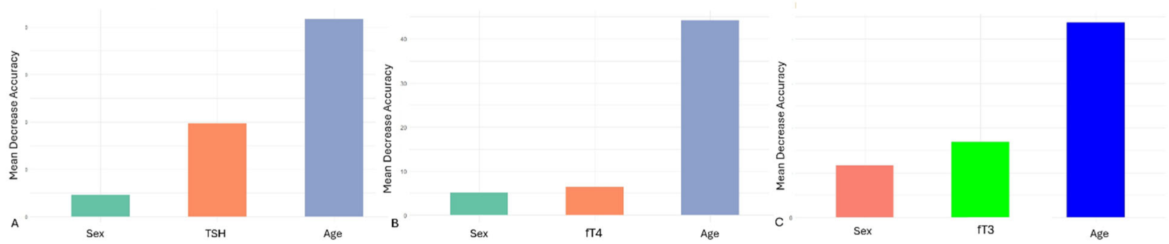

Figure S4. Assessment of feature importance for TSH (A), fT4 (B), and fT3 (C) clusters using the random forest algorithm with age and gender as predictive variables. Abbreviations: fT3, free triiodothyronine; fT4, free thyroxine; TSH, thyroid stimulating hormone.

Table S16. Reference intervals for TSH, fT4, and fT3 obtained using the refineR method.

Analyte (Unit)	Age	Median (1st and 3rd quartiles)	N (Female/Male)	Lower Limit (90% CI)	Upper Limit (90% CI)
TSH (mIU/L)	18-104	1.85 (1.26-2.73)	3426 / 2403	0.64 (0.46–0.69)	5.46 (3.39–5.75)
fT4 (pmol/L)	18-104	14.93 (13.64- 16.47)	2551 / 1905	11.70 (11.30–11.90)	19.80 (19.00–19.80)
fT3 (pmol/L)	18-98	5.22 (4.78-5.70)	1080 / 953	4.06 (3.94–4.23)	6.70 (6.39–6.76)

Abbreviations: CI, confidence interval; fT3, free triiodothyronine; fT4, free thyroxine; TSH, thyroid stimulating hormone.

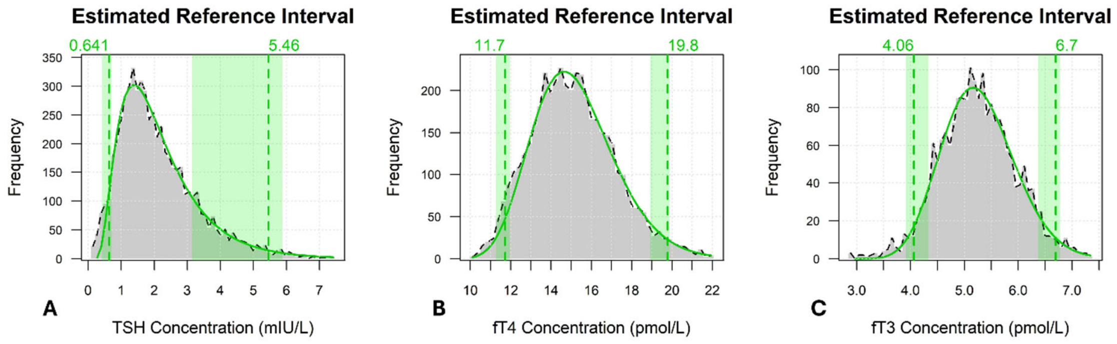

Figure S5. Estimation of reference intervals using the refineR indirect method. The histogram represents the frequency distribution of TSH, fT4 and fT3 concentrations in the study population. The solid green line indicates the modeled non-pathological distribution estimated by the refineR algorithm. The shaded green area and the vertical dashed lines define the calculated reference intervals for TSH (A), fT4 (B), and fT3 (C). Abbreviations: fT3, free triiodothyronine; fT4, free thyroxine; TSH, thyroid stimulating hormone.

Table S17. Reference intervals for TSH, fT4, and fT3 obtained using the reflimR method.

Analyte (Unit)	Age	Median (1st and 3rd quartiles)	N (Female/Male)	Lower Limit (90% CI)	Upper Limit (90% CI)
TSH (mIU/L)	18 - 104	1.85 (1.26-2.73)	3426 / 2403	0.63 (0.61–0.65)	5.69 (5.48–5.82)
fT4 (pmol/L)	18-104	14.93 (13.64- 16.47)	2551 / 1905	10.09 (10.81–11.07)	18.80 (18.62–18.87)
fT3 (pmol/L)	18-98	5.22 (4.78-5.70)	1080 / 953	3.92 (3.87–3.99)	6.54 (6.46–6.59)

Abbreviations: CI, confidence interval; fT3, free triiodothyronine; fT4, free thyroxine; TSH, thyroid stimulating hormone.

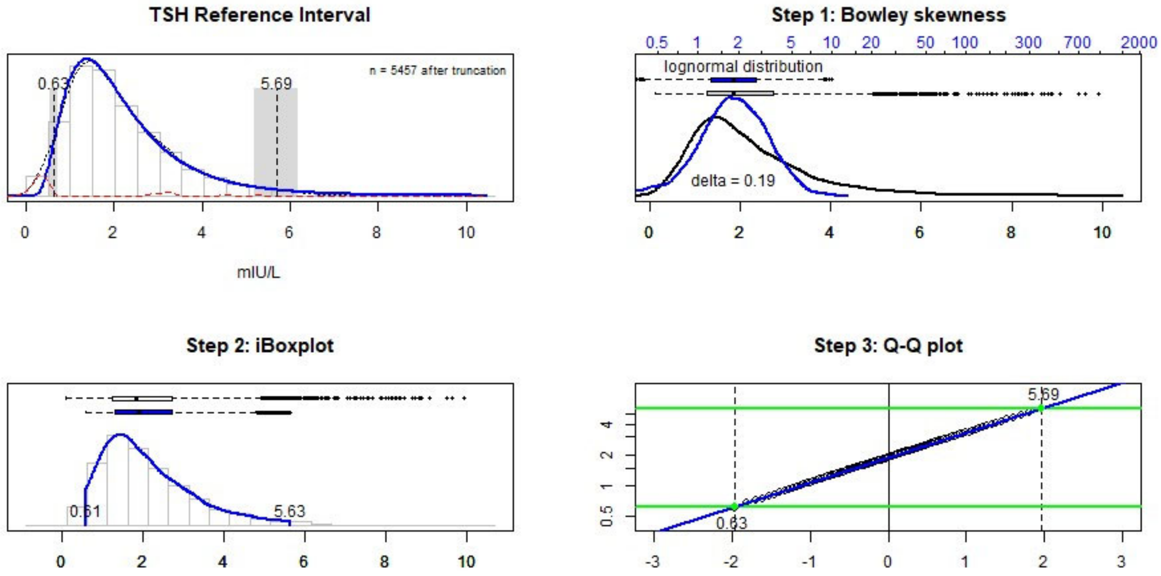

Figure S6. Reference interval estimation plots for TSH derived via the reflimR algorithm. Abbreviations: Q-Q plot, quantile – quantile plot; TSH, thyroid stimulating hormone.

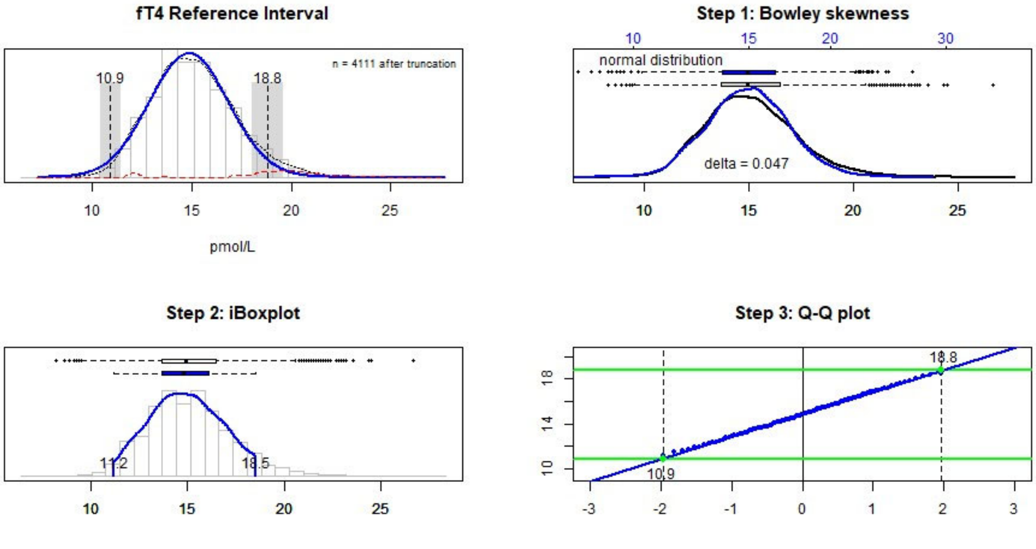

Figure S7. Reference interval estimation plots for fT4 derived via the reflimR algorithm. Abbreviations: fT4, free thyroxine; Q-Q plot, quantile – quantile plot.

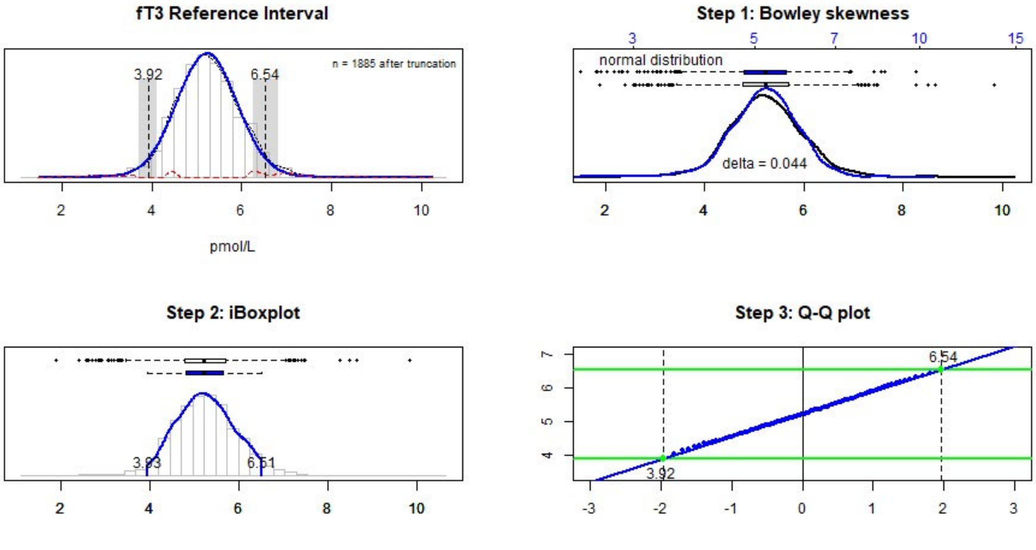

Figure S8. Reference interval estimation plots for fT3 derived via the reflimR algorithm. Abbreviations: fT3, free triiodothyronine; Q-Q plot, quantile – quantile plot.

Table S18. Reference intervals for TSH, fT4, and fT3 calculated using the Bhattacharya method.

Analyte (Unit)	Age	Median (1st and 3rd quartiles)	N (Female/Male)	Lower Limit (90% CI)	Upper Limit (90% CI)
TSH (mIU/L)	18 - 104	1.85 (1.26-2.72)	3386 / 2372	0.50 (0.48-0.51)	5.17 (5.10-5.27)
fT4 (pmol/L)	20-79	14.93 (13.64- 16.47)	2528 / 1885	11.33 (11.20–11.45)	19.43 (19.18–19.56)
fT3 (pmol/L)	24-51	5.22 (4.78-5.70)	1053 / 941	3.90 (3.86–3.96)	6.71 (6.65–6.77)

Abbreviations: CI, confidence interval; fT3, free triiodothyronine; fT4, free thyroxine; TSH, thyroid stimulating hormone.

Table S19. Reference intervals for TSH, fT4, and fT3 calculated using the nonparametric percentile method.

Analyte (Unit)	Age	Median (1st and 3rd quartiles)	N (Female/Male)	Lower Limit (90% CI)	Upper Limit (90% CI)
TSH (mIU/L)	18 - 104	1.85 (1.26-2.72)	3386 / 2372	0.49 (0.47-0.53)	5.12 (4.99-5.22)
fT4 (pmol/L)	20-79	14.93 (13.64- 16.47)	2528 / 1885	11.58 (11.45–11.58)	19.56 (19.43–19.82)
fT3 (pmol/L)	24-51	5.22 (4.78-5.70)	1053 / 941	3.99 (3.92–4.09)	6.62 (6.56–6.71)

Abbreviations: CI, confidence interval; fT3, free triiodothyronine; fT4, free thyroxine; TSH, thyroid stimulating hormone.

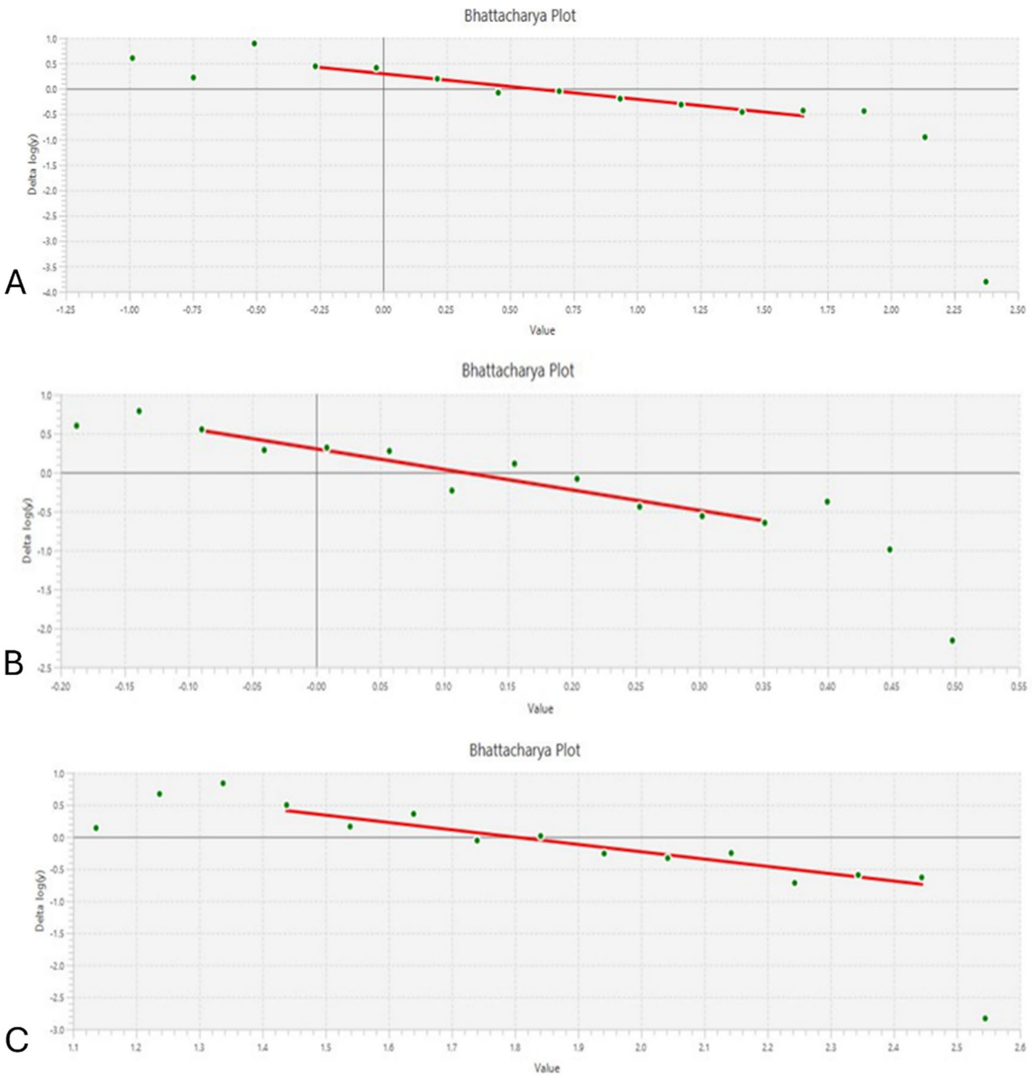

Figure S9. Bhattacharya plots for the estimation of non-pathological distributions of TSH (A), fT4 (B), and fT3 (C). The Bhattacharya plots illustrate the relationship between the class midpoints (Value) and the differences in the logarithms of the frequencies [Delta log(y)]. The solid red lines represent the linear regression fitted to the central part of the distribution, which identifies the gaussian component of the non-pathological population. The points outside the linear trend indicate potential outliers or pathological subpopulations excluded from the final reference interval estimation. Units for the analytes are mIU/L for TSH, ng/dL for fT4, and pg/mL for fT3. Abbreviations: fT3, free triiodothyronine; fT4, free thyroxine; TSH, thyroid stimulating hormone.

Table S20. Diagnostic classification rates (%) based on reference intervals derived from different calculation methods for daily and monthly TSH and fT4 results.

Method	Percentage of daily/monthly change (%)				
	Hyperthyroidism	Hypothyroidism	Euthyroidism	Subclinical Hyperthyroidism	Subclinical Hypothyroidism
Manufacturer	2.2 / 2.2	2.2 / 0.9	83.3 / 84.7	10.0 / 9.0	2.2 / 3.1
Bhattacharya	3.9 / 4.7	2.8 / 1.0	80.0 / 80.5	11.1 / 9.8	2.2 / 3.9
RefineR	4.4 / 4.6	2.2 / 1.1	75.0 / 78.4	16.1 / 12.9	2.2 / 3.0
ReflimR	6.1 / 5.9	2.2 / 0.7	75.0 / 78.8	14.4 / 12.0	2.2 / 2.7
Nonparametric	3.9 / 4.6	2.8 / 1.1	80.6 / 80.7	10.6 / 9.6	2.2 / 3.9
Machine Learning*	4.0 / 2.2	2.9 / 1.7	80.0 / 80.3	10.29 / 8.37	2.86 / 5.14
Harris-Boyd*	4.57 / 4.54	2.86 / 1.8	80.0 / 80.0	9.71 / 8.62	2.86 / 5.06

*Test results were categorized based on the corresponding subgroup-specific reference intervals.
Abbreviations: fT4, free thyroxine; TSH, thyroid stimulating hormone.

Table S21. Number and percentage (%) of individuals with reclassified diagnoses for daily and monthly TSH and fT4 results, comparing manufacturer-provided intervals with reference intervals obtained via nonparametric percentile, Bhattacharya, refineR, and reflimR estimation methods, as well as machine learning and Harris-Boyd partitioning strategies.

Method	Daily Results (N=181) Reclassification Rate (%) / N	Monthly Results (N=4,888) Reclassification Rate (%) / N
Nonparametric	3.89 / 7	6.24 / 305
Bhattacharya	4.44 / 8	6.38 / 312
RefineR	9.44 / 17	8.53 / 417
ReflimR	10.00 / 18	10.29 / 503
Machine Learning*	4.44 / 8	5.30 / 259
Harris-Boyd*	5.00 / 9	5.49 / 268

*Test results were categorized based on the corresponding subgroup-specific reference intervals.
Abbreviations: fT4, free thyroxine; TSH, thyroid stimulating hormone.

Table S22. Lower and upper limits obtained by applying all reference interval estimation methods to subgroups derived from unsupervised machine learning and conventional approaches.

Method	Age Group	TSH Lower	TSH Upper	fT4 Lower*	fT4 Upper*
**ML + refineR**	1	0.69	4.79	11.70	21.00
**ML + refineR**	2	0.65	3.71	10.20	19.50
**ML + refineR**	3	0.44	3.11	11.10	17.30
**ML + refineR**	4	0.20	3.67	11.00	19.90
**ML + refineR**	5	0.31	3.06	10.10	19.70
**ML + refineR****	6	0.32	4.6	11.10	20.7
**Conventional + refineR**	1	0.69	4.79	10.70/ 11.50*	18.60/ 19.90*
**Conventional + refineR**	2	0.67	3.76	10.70/ 11.50*	18.60/ 19.90*
**Conventional + refineR**	3	0.44	3.11	10.70/ 11.50*	18.60/ 19.90*
**Conventional + refineR**	4	0.20	3.67	10.70/ 11.50*	18.60/19.90*
**Conventional + refineR**	5	0.39	3.30	10.70/ 11.50*	18.60/ 19.90*
**Conventional + refineR**	6	0.32	4.6	10.70/ 11.50*	18.60/ 19.90*
**ML + Nonparametric**	1	0.68	5.15	11.71	19.43
**ML + Nonparametric**	2	0.55	5.09	11.71	19.56
**ML + Nonparametric**	3	0.53	4.76	11.45	19.18
**ML + Nonparametric**	4	0.45	4.96	11.45	19.05
**ML + Nonparametric**	5	0.36	5.14	11.33	19.43
**ML + Nonparametric**	6	0.41	5.67	11.33	19.43
**Conventional + Nonparametric**	1	0.68	5.15	11.45/ 11.71*	19.18/ 20.08*
**Conventional + Nonparametric**	2	0.59	5.06	11.45/11.71*	19.18/ 20.08*
**Conventional + Nonparametric**	3	0.53	4.75	11.45/ 11.71*	19.18/ 20.08*
**Conventional + Nonparametric**	4	0.45	4.95	11.45/ 11.71*	19.18/ 20.08*
**Conventional + Nonparametric**	5	0.38	5.14	11.45/ 11.71*	19.18/ 20.08*
**Conventional + Nonparametric**	6	0.43	5.67	11.45/ 11.71*	19.18/2 0.08*
**Conventional + reflimR**	1	0.74	5.40	10.81/ 11.20*	18.15/ 19.56*
**Conventional + reflimR**	2	0.73	5.11	10.81/ 11.20*	18.15/ 19.56*
**Conventional + reflimR**	3	0.54	6.46	10.81/ 11.20*	18.15/ 19.56*
**Conventional + reflimR**	4	0.65	5.54	10.81/ 11.20*	18.15/ 19.56*
**Conventional + reflimR**	5	0.49	5.24	10.81/ 11.20*	18.15/19.56*
**Conventional + reflimR**	6	0.44	6.14	10.81/ 11.20*	18.15/ 19.56*
**ML + reflimR**	1	0.74	5.40	11.20	19.18
**ML + reflimR**	2	0.73	5.11	10.55	18.28
**ML + reflimR**	3	0.54	6.46	10.94	18.53
**ML + reflimR**	4	0.65	5.54	10.94	18.79
**ML + reflimR**	5	0.48	5.36	10.68	18.92
**ML + reflimR****	6	0.43	6.51	10.81	19.95
**ML + Bhattacharya**	1	0.63	4.18	11.68	18.56
**ML + Bhattacharya**	2	0.45	4.67	11.42	19.23
**ML + Bhattacharya**	3	0.40	4.32	11.09	20.59
**ML + Bhattacharya**	4	0.43	4.58	11.89	19.68
**ML + Bhattacharya**	5	0.38	4.20	11.08	19.30
**ML + Bhattacharya**	6	0.32	4.83	9.67	22.60

**Conventional+ Bhattacharya**	1	0.63	4.18	11.09/ 11.89*	18.97/ 18.86*
**Conventional+ Bhattacharya**	2	0.54	4.56	11.09/ 11.89*	18.97/ 18.86*
**Conventional+ Bhattacharya**	3	0.40	4.32	11.09/ 11.89*	18.97/ 18.86*
**Conventional+ Bhattacharya**	4	0.43	4.58	11.09/ 11.89*	18.97/ 18.86*
**Conventional+ Bhattacharya**	5	0.32	4.15	11.09/ 11.89*	18.97/ 18.86*
**Conventional+ Bhattacharya**	6	0.42	4.94	11.09/ 11.89*	18.97/ 18.86*
**refineR**	All Population	0.64	5.46	11.70	19.80
**Nonparametric**	All Population	0.49	5.12	11.58	19.56
**Bhattacharya**	All Population	0.50	5.17	11.33	19.43
**reflimR**	All Population	0.63	5.69	10.09	18.80

** For Conventional (Harris) subgroups, fT4 partitioning limits represent Female / Male splits respectively. ** To achieve the robust sample size required for indirect reference interval estimation methods (refineR and reflimR), the reference population was expanded ($n = 614$ for TSH and $n = 581$ for free T_4).*

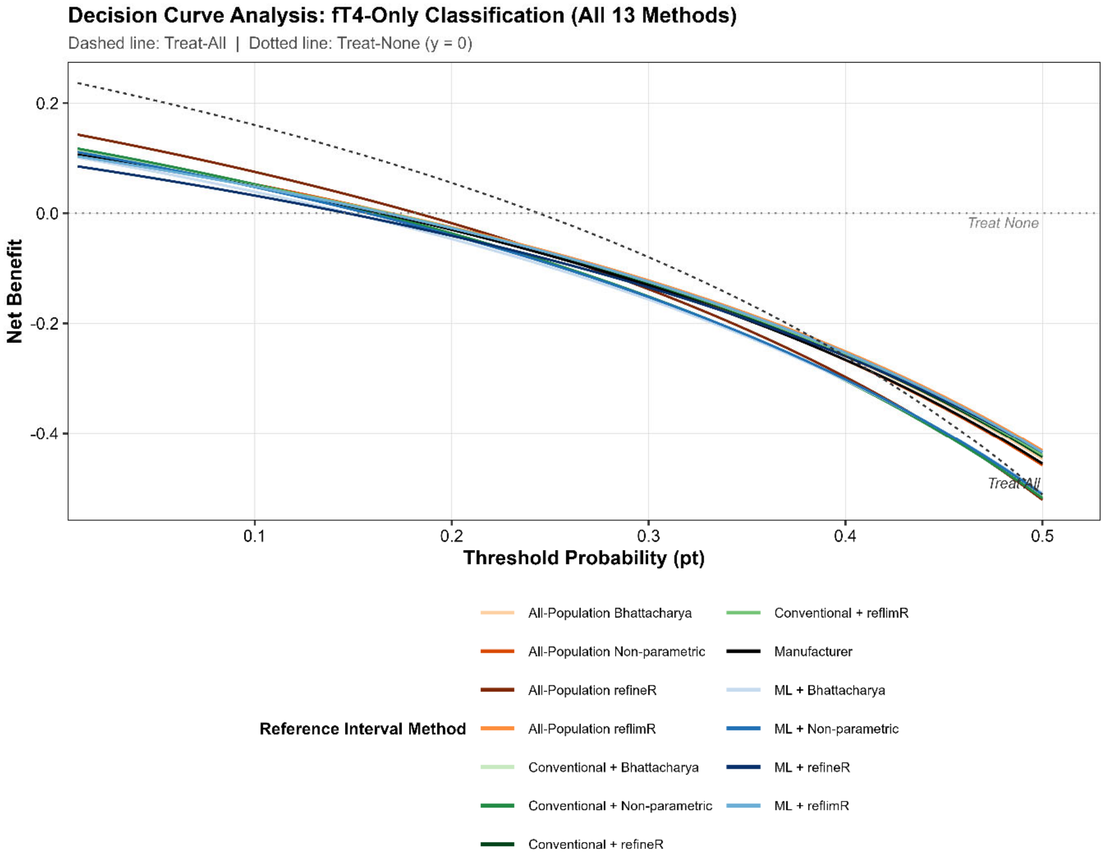

Figure S10. Decision curve analysis (DCA) evaluating the clinical utility of the 13 reference interval methods for free thyroxine (fT4).

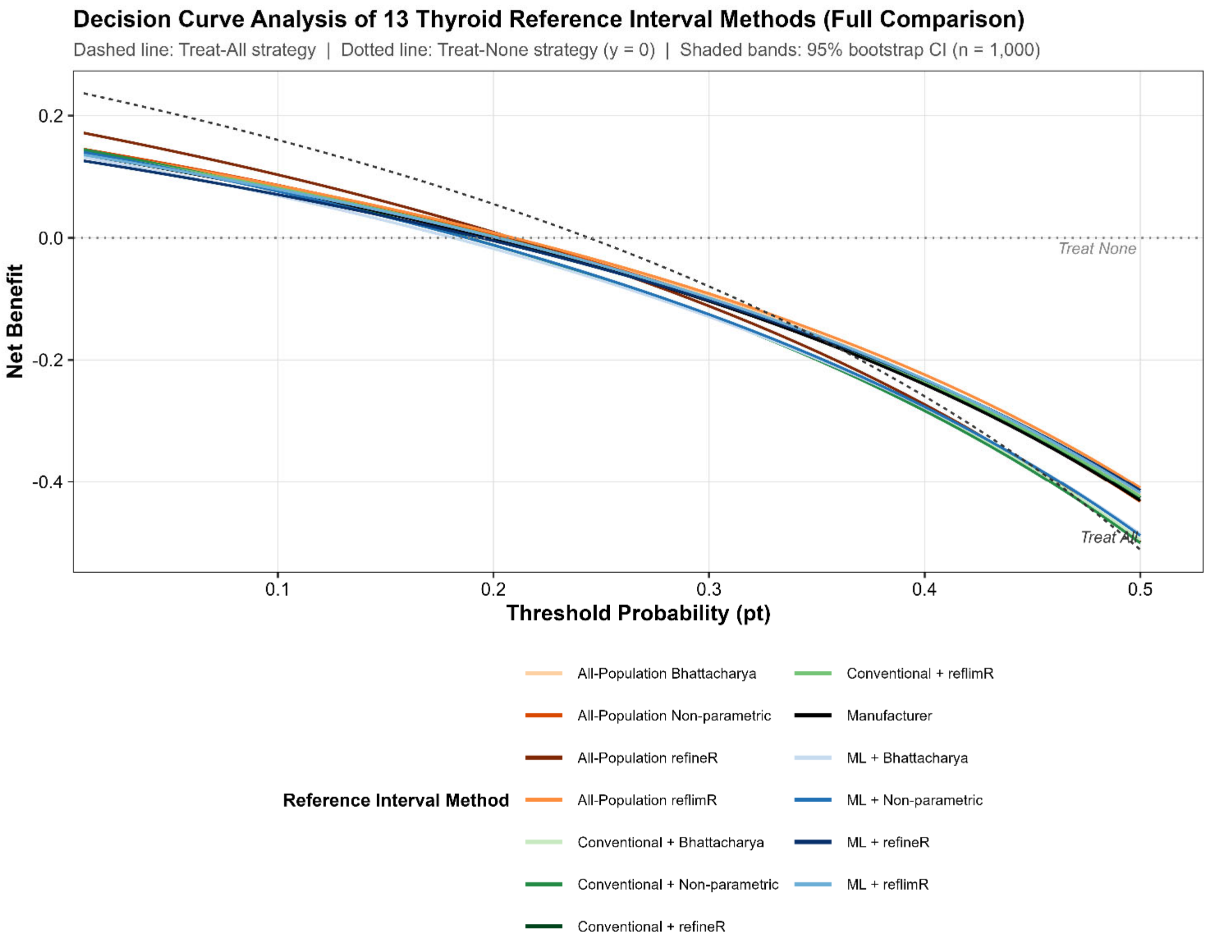

Figure S11. Decision curve analysis (DCA) evaluating the clinical utility of the 13 reference interval methods for TSH and fT4.

Table S23. Diagnostic performance based on TSH and fT4 classification (NHANES, N = 2297)

Method	Sensitivity, % (95% CI)	Specificity, % (95% CI)	PPV, %	NPV, %	Youden Index
All-Pop. reflimR	60.4 (56.2–64.5)	26.2 (24.2–28.3)	20.9	67.2	-0.134
All-Pop. Bhattacharya	61.1 (57.0–65.2)	24.4 (22.4–26.5)	20.7	66.0	−0.145
All-Pop. Nonparametric	61.9 (57.7–65.9)	22.8 (20.8–24.8)	20.6	64.9	−0.154
Conventional + reflimR	59.2 (55.0–63.3)	24.7 (22.7–26.8)	20.3	65.2	−0.161
ML + reflimR	57.9 (53.7–62.1)	25.9 (23.9–28.1)	20.2	65.6	−0.161
Conventional + refineR	59.2 (55.0–63.3)	24.4 (22.4–26.5)	20.2	64.9	−0.164
All-Pop. refineR	73.1 (69.2–76.7)	10.4 (9.0–11.9)	20.9	54.4	−0.165
Manufacturer	57.9 (53.7–62.1)	24.2 (22.2–26.3)	19.8	64.0	−0.179
ML + refineR	53.8 (49.6–58.0)	27.8 (25.7–29.9)	19.4	65.0	−0.184
ML + Nonparametric	60.1 (55.9–64.2)	16.0 (14.3–17.8)	18.8	55.3	−0.240
Conventional + Nonparametric	61.9 (57.7–65.9)	13.8 (12.2–15.5)	18.8	52.8	−0.244
Conventional + Bhattacharya	60.8 (56.6–64.8)	14.8 (13.2–16.6)	18.7	53.9	−0.244
ML + Bhattacharya	56.5 (52.3–60.7)	17.5 (15.7–19.3)	18.1	55.4	−0.260

Abbreviations: PPV, positive predictive value; NPV, negative predictive value; CI, confidence interval; ML, machine learning (data-driven partitioning); All-Pop., All-Population (unpartitioned). Methods are ordered by descending Youden index within each table. N = 561 patients, N = 1736 reference individuals; disease prevalence = 24.4%.

Table S24. Diagnostic performance based on fT4-only classification (NHANES, N = 2,297)

Method	Sensitivity, % (95% CI)	Specificity, % (95% CI)	PPV, %	NPV, %	Youden Index
All-Pop. reflimR	45.1 (40.9–49.3)	28.3 (26.2–30.5)	16.9	61.5	−0.266
Conventional + reflimR	45.6 (41.5–49.9)	27.1 (25.0–29.2)	16.8	60.6	−0.273
All-Pop. Bhattacharya	47.1 (42.9–51.3)	25.6 (23.6–27.8)	17.0	60.0	−0.273
ML + reflimR	44.6 (40.4–48.8)	28.1 (26.0–30.3)	16.7	61.1	−0.273
All-Pop. refineR	61.3 (57.1–65.4)	11.2 (9.8–12.8)	18.2	47.3	−0.274
Conventional + refineR	45.1 (40.9–49.3)	26.8 (24.7–28.9)	16.6	60.2	−0.281
All-Pop. Nonparametric	47.8 (43.6–52.0)	23.9 (21.9–26.0)	16.9	58.6	−0.283
Manufacturer	45.8 (41.6–50.0)	24.9 (22.9–27.0)	16.5	58.8	−0.292
ML + refineR	37.1 (33.1–41.2)	30.5 (28.3–32.7)	14.7	60.0	−0.325
Conventional + Nonparametric	50.8 (46.6–55.0)	15.0 (13.4–16.8)	16.2	48.6	−0.342
Conventional + Bhattacharya	49.2 (45.0–53.4)	15.9 (14.2–17.7)	15.9	49.2	−0.349
ML + Nonparametric	48.1 (43.9–52.4)	16.8 (15.0–18.6)	15.7	50.0	−0.351
ML + Bhattacharya	44.0 (39.9–48.2)	18.4 (16.6–20.3)	14.9	50.5	−0.375

Abbreviations: PPV, positive predictive value; NPV, negative predictive value; CI, confidence interval; ML, machine learning (data-driven partitioning); All-Pop., All-Population (unpartitioned). Methods are ordered by descending Youden index within each table. N = 561 patients, N = 1,736 reference individuals; disease prevalence = 24.4%.
